# Statins ameliorate cholesterol-induced inflammation and improve AQP2 expression by inhibiting NLRP3 activation in the kidney

**DOI:** 10.7150/thno.49603

**Published:** 2020-08-20

**Authors:** Yonglun Kong, Weijing Feng, Xiaoduo Zhao, Puhua Zhang, Suchun Li, Zhijian Li, Yu Lin, Boen Liang, Chunling Li, Weidong Wang, Hui Huang

**Affiliations:** 1 Department of Cardiology, The Eighth Affiliated Hospital, Sun Yat-sen University, Shenzhen 518033, China.; 2 Department of Pathophysiology, Zhongshan School of Medicine, Sun Yat-sen University, Guangzhou, 510080, China.; 3 Guangdong Provincial Key Laboratory of Malignant Tumor Epigenetics and Gene Regulation, Department of Cardiology, RNA Biomedical Institute, Sun Yat-sen Memorial Hospital of Sun Yat-sen University, Guangzhou, 510120, China.; 4 Department of Cardiology, Laboratory of Heart Center, Zhujiang Hospital, Southern Medical University, Guangzhou, 510280, China.; 5 Key Laboratory of Nephrology, National Health Commission and Guangdong Province, Department of Nephrology, The First Affiliated Hospital, Sun Yat-sen University, Guangzhou, 510080, Chinaa.; 6 Department of Pathology, Zhujiang Hospitial, Southern Medical University, Guangzhou, 510282, China.; 7 Institute of Hypertension, Zhongshan School of Medicine, Sun Yat-sen University, Guangzhou, 510080, China.

**Keywords:** cholesterol, statins, AQP2, IL-1β, ASC

## Abstract

**Background:** Chronic kidney diseases (CKD) are usually associated with dyslipidemia. Statin therapy has been primarily recommended for the prevention of cardiovascular risk in patients with CKD; however, the effects of statins on kidney disease progression remain controversial. This study aims to investigate the effects of statin treatment on renal handling of water in patients and in animals on a high-fat diet.

**Methods:** Retrospective cohort patient data were reviewed and the protein expression levels of aquaporin-2 (AQP2) and NLRP3 inflammasome adaptor ASC were examined in kidney biopsy specimens. The effects of statins on AQP2 and NLRP3 inflammasome components were examined in *nlrp3^-/-^* mice, 5/6 nephroectomized (5/6Nx) rats with a high-fat diet (HFD), and *in vitro*.

**Results:** In the retrospective cohort study, serum cholesterol was negatively correlated to eGFR and AQP2 protein expression in the kidney biopsy specimens. Statins exhibited no effect on eGFR but abolished the negative correlation between cholesterol and AQP2 expression. Whilst *nlrp3^+/+^* mice showed an increased urine output and a decreased expression of AQP2 protein after a HFD, which was moderately attenuated in *nlrp3* deletion mice with HFD. In 5/6Nx rats on a HFD, atorvastatin markedly decreased the urine output and upregulated the protein expression of AQP2. Cholesterol stimulated the protein expression of NLRP3 inflammasome components ASC, caspase-1 and IL-1β, and decreased AQP2 protein abundance *in vitro*, which was markedly prevented by statins, likely through the enhancement of ASC speck degradation via autophagy.

**Conclusion:** Serum cholesterol level has a negative correlation with AQP2 protein expression in the kidney biopsy specimens of patients. Statins can ameliorate cholesterol-induced inflammation by promoting the degradation of ASC speck, and improve the expression of aquaporin in the kidneys of animals on a HFD.

## Introduction

Hyperlipidemia has been hypothesized to play an important role in the progression of kidney injury 1, at least partially, by causing deleterious renal lipid disturbance. Chronic kidney disease (CKD) is a common disorder that is associated with an increased risk of a number of complications, such as cardiovascular disease and end-stage renal disease (ESRD). The incidence of CKD has been shown to be associated with increased levels of plasma triglyceride and low-density lipoprotein cholesterol (LDL-c), as well as decreased levels of HDL cholesterol in epidemiologic studies 2-4. Dyslipidemia (e.g. hypercholesterolemia), hypertension and diabetes mellitus are common in patients with CKD. Based on available randomized trial data of lipid-lowering therapies, statin therapy has been recommended by the KDIGO for lipid management in CKD 5.

It has been shown that lipids may cause injuries in both glomerular and tubular cells which then promote renal disease progression 6, 7. Consistently, several studies 8-11 including meta-analyses 12, 13 have shown that treatment with statins, which are HMG-CoA reductase inhibitors, can attenuate proteinuria and preserve renal function in a manner that is independent of other variables. A systematic review coupled with meta-analysis has revealed that, in adult patients not receiving dialysis, statins can modestly decrease proteinuria and attenuate the decline of GFR 14. In animal models, statins have been found to preserve renal function in 5/6 nephrectomized (5/6Nx) rats, which may indicate a protective role of statins in preserving compromised renal function 15, 16. Statin therapy has been shown to clearly lower the risk of cardiovascular disease and the mortality in patients with CKD 17, 18. However, statin therapy appears not reduce the incidence of ESRD or the annual rate of decline in eGFR 19 and has little or no effect on patients receiving dialysis 18. High-efficacy statins, such as atorvastatin have been shown to be associated with an increased hazard for developing severe renal failure compared to that of low-efficacy statins 20, 21. Therefore, the effects of statins on the progression of kidney disease remain controversial.

It is likely that there are different lipid accumulation mechanisms that affect the glomerular and tubular structures and functions in the kidneys. Although studies on GFR and proteinuria are undoubtedly important 22, investigations on the specific mechanisms of lipid-induced renal tubular dysfunction are especially required. Arginine vasopressin (AVP, also called antidiuretic hormone ADH) plays a key role in urine concentration and body water homeostasis. Aquaporin-2 (AQP2) is an important AVP-regulated water channel protein localized in the principal cells of collecting ducts in the kidneys. An increased level of circulating AVP induces protein expression, phosphorylation, and intracellular trafficking of AQP2, leading to water transport across the epithelium in the collecting ducts. Statins have been shown to induce apical expression of AQP2 in the principal cells 23-25. Interestingly, studies have shown that, an increased level of vasopressin in people with obesity 26, 27 and it was associated with insulin resistance, adiposity and metabolic syndrome 28. An early study has demonstrated that hydration, which is supposed to inhibit vasopressin secretion, can ameliorated progression of CKD induced by 5/6Nx in rats 29. However, CKD is usually associated with the retention of water and sodium, as well as the expansion of fluid volume, which may lead to an increased cardiac preload and blood pressure, coupled with dyslipidemia and a higher risk of cardiovascular disease development. Impaired natriuresis, which is one of the obesity-related tubular alterations of the kidneys, has been presumed to be able to at least partially contribute to the maintenance or development of hypertension 30. As statins have been widely used in CKD for preventing the cardiovascular risk, it is necessary to investigate whether statins can affect the metabolism of water and sodium in the kidneys.

Several molecular mechanisms, including the generation of reactive oxygen species, multiple organelle damage, release of proinflammatory and profibrotic factors, and lipid-induced apoptosis, appear to mediate cellular dysfunction and injury caused by lipid accumulation in renal tubular segments 22. A recent study has shown that a high-fat diet can cause lysosomal system dysfunction and lipid metabolism alteration characterized by the accumulation of cholesterol and phospholipid in tubular epithelial cells of the kidneys 31. Interestingly, HMG-CoA reductase has been found to be expressed in collecting ducts and in proximal tubular epithelial cells, and the expression can be greatly increased by high-fat diet, indicating a potential role of tubular epithelial cells in cholesterol regulation in the kidney 31. The over-accumulation of intracellular cholesterol is cytotoxic, and it may stimulate an adaptive phospholipid biosynthesis 32 and immune response. Nod-like receptor protein 3 (NLRP3), an innate immune receptor is a cytosolic protein that induces inflammation 33. Upon detecting danger signals, NLRP3 recruits and assembles with adaptor ASC and caspase-1, which results in activation of caspase-1 and maturation of IL-1β (and IL-18), inducing inflammatory response. NLRP3 inflammasome has been shown to be actively involved in intracellular cholesterol homeostasis and cholesterol accumulation in the renal epithelial cells 34.

Several key renal sodium transporters and channels located in the tubular epithelial cells are critically involved in the reabsorption and excretion of sodium and water in the kidneys. The expression of these proteins is tightly regulated by hormones such as vasopressin and aldosterone, depending on status of body sodium and water. Local factors in the kidneys, including pro-inflammatory cytokines (e.g. IL-1β) have also been shown to affect the protein expression of aquaporins in several kidney injury models 35-37. Although an earlier study has demonstrated downregulated levels of sodium transporters and aquaporins in the tubular segments and collecting ducts of diabetic Zucker rats, the underlying molecular mechanism remains elusive 38. It is not fully understood whether abnormal lipid accumulation in the tubular epithelial cells can affect renal handling of water and sodium.

In the present study, the effects of dyslipidemia, particularly hypercholesterolemia, and statin treatment on renal handling of water were investigated in a retrospective clinical cohort study. The potential underlying molecular mechanisms were also examined in NLRP3 inflammasome deficient mice and CKD animals on a high-fat diet.

## Methods and Materials

### Study design, participants, and data collection

All hospitalized patients diagnosed with or without CKD in the affiliated hospital of Sun Yat-sen University from January 2012 to September 2019 were retrospectively reviewed: group A, a total of 231 subjects had 24 h urine and plasma lipid levels assayed; group B, subjects who treated with (n = 50) or without (n = 84) atorvastatin (ATO) had estimated glomerular filtration rate (eGFR) and plasma lipid levels assayed; group C, subjects who treated with (n = 9) or without (n = 6) atorvastatin had kidney biopsy for diagnosis. Patients who met the following criteria were excluded: acute myocardial infarction, stroke, cirrhosis, renal carcinoma, polycystic kidney disease, recipient of organ transplant, and acute inflammatory or infection. The hospital ethic committee approved this study and waived the need for patients' consent. These patients' right of privacy has been well protected in clinical diagnosis and treatment. The study was conducted in accordance with the Declaration of Helsinki (2013).

A baseline medical history questionnaire was used to obtain information on demographic characteristics, previous history of disease, and use of medications. Biochemical parameters, including serum sodium, potassium, fasting plasma glucose, creatinine, urea nitrogen, total cholesterol, and LDL-c were measured using venous plasma samples. All venous plasma samples were measured by a standardized and certified program using an automatic biochemical analyzer (AU5800, BECKMAN COULTER, USA). A 24 h urine specimen was collected and urine volume, urinary sodium, urinary potassium, urinary albumin excretion rate were analyzed using a standardized and certified TBA-120 auto-analyzer (Toshiba Medical Systems, Japan) in the institutional central laboratory. eGFR was calculated using the Chronic Kidney Disease Epidemiology Collaboration equation with modified coefficients for the Chinese population 39. Kidney biopsies were performed under ultrasound guidance using the Trucut biopsy needle. The indications for biopsy were: non-nephrotic proteinuria alone, haematuria and proteinuria, nephrotic range proteinuria, acute renal failure, haematuria alone, and chronic renal failure.

### Animals

All animal procedures were approved by the Animal Care and Use Committee of Sun Yat-sen University (Ethics Committee of ZSSOM on laboratory Animal Care No. 2016-048; Guangzhou, China). Mice with *nlrp3* gene deletion on a background of C57BL/6 were obtained from VIEWSOLID BIOTECH, Beijing, China. For identification of the genotypes, DNA extracted from tails of both *nlrp3^+/+^* and *nlrp3^-/-^* mice were amplified by PCR and then used for agarose gel electrophoresis or DNA sequence analysis. Ten-week-old male C57BL/6 (WT) and *nlrp3^-/-^* mice were enrolled in these experiments. Male Sprague-Dawley rats were divided into three groups: 5/6Nx rats treated with standard laboratory chow; 5/6Nx rats treated with high-fat diet chow; and 5/6Nx rats treated with high-fat diet chow and atorvastatin (20 mg/kg BW/day). The mice and rats were fed with a standard chow (10% of total calories from fat; cholesterol 18 mg/kg) or a high-fat diet (60% of total calories from fat; cholesterol 300.8 mg/kg, Guangdong Medical Lab Animal Center, China) for 12 wk. The diets are otherwise identical in their protein, mineral, and ion content. The animals were housed in a room with 12 h light/dark cycle with a temperature of 25 °C. Mice or rats were placed in metabolic cages for 24 h urine collection at week 12. On the sacrifice day, all animals were anesthetized with pentobarbital, and the kidneys were rapidly excised to perform biochemical and histological examinations. All animal procedures were in accordance with the policies of the Animal Care and Use Committee, Sun Yat-Sen University, and conformed to the Guide for the Care and Use of Laboratory Animals of the National Institute of Health in China.

### IMCD tubule preparation and treatments

Rat IMCD suspensions were prepared as previously described 40. Primary IMCD cells were pretreated with or without atorvastatin (10 μM or 20 μM) or dDAVP (10^-7^ M) for 30 mins, and then incubated with cholesterol (200 ng/mL) or a vehicle for 6 h. After the incubation, protein was collected in radioimmunoprecipitation buffer with proteinase cocktails, and the samples were used for immunoblotting as described previously. The experiment was repeated three times.

### mpkCCD preparation and treatments

To examine the effects of simvastatin on cholesterol induced inflammation activation, immortalized mouse cortical collecting duct cell line (mpkCCD cells) were seeded on 6 wells plates and then incubated in serum-free medium for 12 h before cholesterol and simvastatin treatment. Then, cholesterol (C4591 Sigma, USA) for the last 24 h. To exase the effects of simvastatin on inflammation components during cholesterol treatment, simvastatin were added for the last 24 h.

### Immunofluorescence studies

For immunofluorescence, rat IMCD suspensions were treated with hyperosmotic DMEM/F12 medium containing 10% FBS, 100 U/ml penicillin Gm and 100 U/ml streptomycin sulfate. In order to maintain AQP2 expression, the cells were grown on 24 mm plates and 8cr-cAMP was added. When at 70%-80% confluence, the cells were switched to hyperosmotic medium without FBS for 6 h before experiment began. After treatment above, IMCD cells were washed twice with cold PBS and fixed with 4% paraformaldehyde. Immunofluorescence was performed for examining AQP2 expression and trafficking in IMCD cells.

mpkCCD cells cultured on 35 mm glass were subjected to the confocal analysis after fixed in 4% paraformaldehyde and permeabilized with 0.5% Triton X-100 for 15 mins at room temperature. After being blocked with 10% goat serum in TBST at room temperature for 1 h, cells were incubated with ASC antibody (1:400, sc-514414 Santa Cruz Biotechnology, USA) and SQSTM1 (1:400, 5114 Cell signaling technology, USA) antibody overnight at 4 °C. Cells were washed and then incubated with Alexa Fluor-488-conjugated goat anti-mouse secondary antibody and Alexa Fluor-555-conjugated goat anti-rabbit secondary antibody for 1 h at room temperature. The nuclei were counterstained in blue with 4, 6-diamidino-2-phenylindole (DAPI).

### Western blotting and coimmunoprecipitation (IP) studies

IMCD, mouse cortical collecting duct principal cell line mpkCCD cells or renal tissue were lysed in protein lysis buffer for 15 mins on ice before protein was extracted. Immunoblotting was performed with primary antibodies against aquaporin-2 (AQP2, 1:3000), aquaporin-3 (AQP3 1:1000) 35, p-Ser256 AQP2 (1:1000, ab111346 Abcam, USA), NLRP3 (1:1000, ab214185 Abcam, USA; 1:1000), Caspase-1 (p20) (1:1000, AB1871 Milipore, USA), ASC (1:1000, sc-514414 Santa Cruz Biotechnology, USA), IL-1β (1:1000, sc-57954 Santa Cruz Biotechnology, USA), SQSTM1 (1:1000, 5114 Cell signaling technology, USA), LC3B (1:1000 2775S Cell signaling technology, USA) followed by the addition of horseradish peroxidase-labelled secondary antibodies. The blots were visualized with ECL detection systems. Densitometric analysis was performed using AlphaEase Software.

The samples subjected to IP assay were incubated with an anti-ASC antibody 1:10 (sc-514414 Santa Cruz Biotechnology, USA) in IP buffer overnight at 4 °C. Protein A-sepharose beads were added to the samples, which incubated for another 12 h. The sample were then washed and resuspended, and Western blotting was performed as described previously.

### Histologic analysis

Paraffin-embedded kidney sections used for IHC studies were dewaxed, rehydrated, and incubated with primary antibodies against aquaporin-2 (AQP2, 1:3000) overnight at 4 °C. The sections were subsequently incubated with secondary antibodies, treated with diaminobenzidine, counterstained with hematoxylin and examined as previously reported 40.

### Quantitative RT-PCR

Total RNA was extracted from the kidney cortex or cultured cells according to the manufacturer's instructions for Trizol reagent (Invitrogen). Total RNA (1,000 ng) was used for reverse transcription using PrimeScript RT Reagent Kit Perfect Real Time Kit (Takara Bio). The cDNA was used for quantitative real-time PCR analysis (qPCR) using SYBR Premix Ex Taq (Perfect Real Time) (Takara Bio). All samples were analyzed in triplicate. The calibrator sample was selected from PBS-treated tissue or cell samples, and GAPDH was used as an internal control. Relative amounts of mRNA were normalized by GAPDH and a control sample and calculated by using the comparative Ct (2-ΔΔCT) (cycle threshold) method. Signals from the control group were assigned a relative value of 1.0. Primers were designed based on previous publications or on the primerbank (supplementary data S1).

### Statistical analysis

Results are presented as the means ± SEM. Data were analyzed by one-way ANOVA and Newman-Keuls tests for multiple comparisons. Statistical significance was accepted at the *p* < 0.05 level.

## Results

### Elevated serum cholesterol levels were associated with increased urine volume and urinary sodium excretion in patients

Baseline characteristics of study participants (Group A, n = 231) by quartile of total cholesterol are presented in Table 1. The correlations between total cholesterol, eGFR, urine volume and sodium excretion were evaluated by linear regression analysis. As shown in Table 2, following parameter adjustment of gender and age, whilst a negative correlation was found between total cholesterol and eGFR (*p =* 0.038, *r =* -0.137), no statistical correlations were observed between serum sodium and serum cholesterol levels. However, total cholesterol was revealed to exhibit a positive correlation individually with increased urinary sodium excretion (*p =* 0.011, *r =* 0.167), and with urine volume (*p =* 0.007, *r =* 0.178).

### Atorvastatin decreased serum cholesterol and LDL-C, but failed to improve eGFR in patients

The lipid profile and eGFR profiles of patients (Group B) with atorvastatin (ATO, n = 50) and the corresponding control group (CTL, n = 84) are shown in Table 3. Patients in the ATO group showed significantly lower serum levels of total cholesterol, LDL-c and Hs-CRP than those in the control group (Table 3). Interestingly, no significant difference in eGFR was found between the two groups (*p >* 0.05). In order to examine the effects of atorvastatin on different stages of CKD, the patients were grouped into three subgroups based on the eGFR (ml/min·per 1.73 m^2^): group a (eGFR ≥ 90), group b (60 ≤ eGFR < 90) and group c (eGFR < 60). As shown in Table 3, whilst atorvastatin decreased the serum levels of total cholesterol, LDL-c and Hs-CRP in patients of all three groups; atorvastatin failed to improve the eGFR levels in all stages of CKD (Table 3). Notably, in group c with an eGFR level of < 60, a slight, insignificant decrease of the eGFR was observed in patients with ATO.

### Elevated serum cholesterol levels in patients were associated with decreased AQP2 protein expression in the kidney biopsy samples

**Figure 1:**
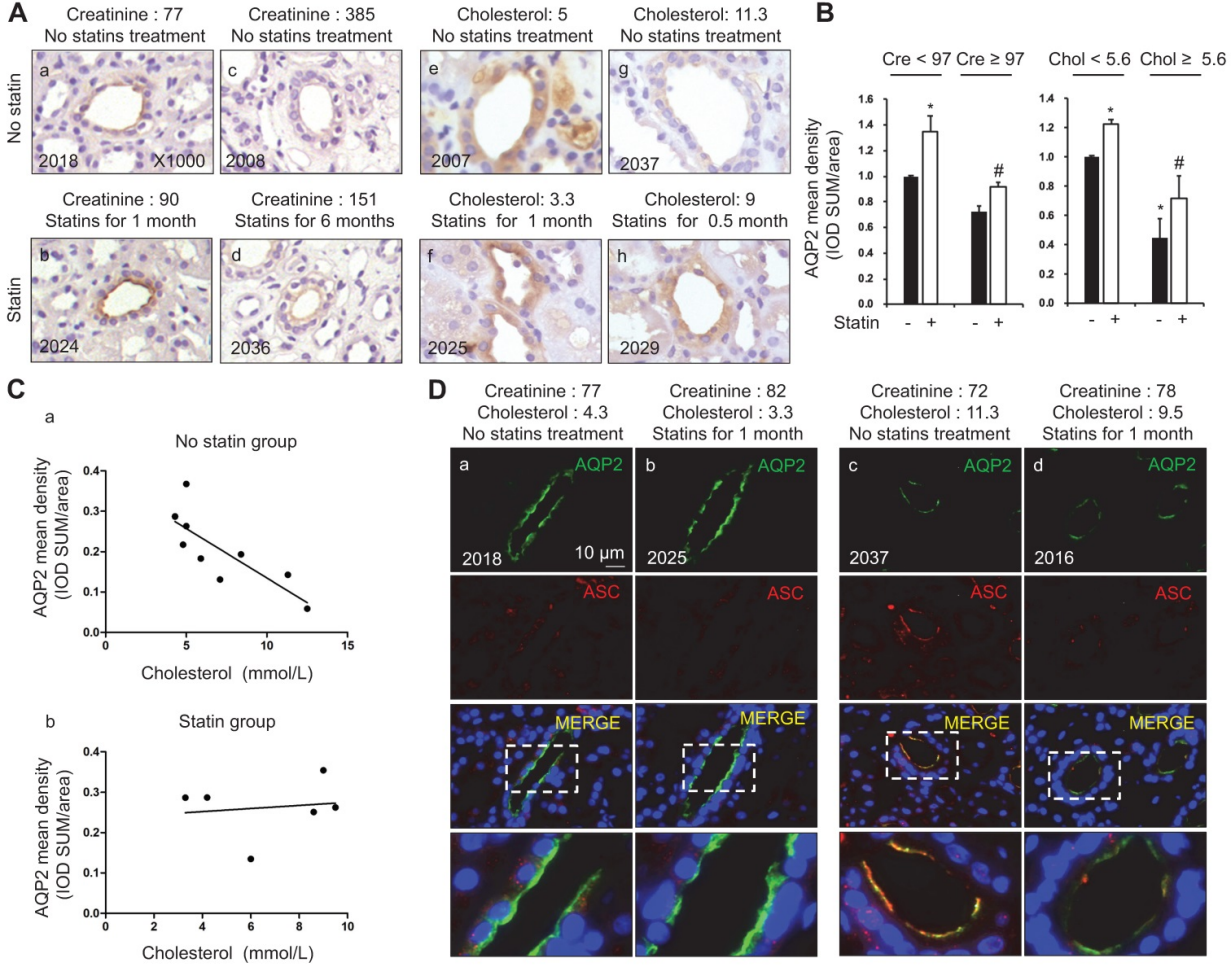
Statins treatment was associated with increased AQP2 protein expression in the kidney biopsy samples of patients with dyslipidemia.** (A)** Statin treatment was associated with increased AQP2 expression in human kidney specimen detected by immunohistochemistry. **(B)** AQP2 mean density (IOD SUM/area) analysis in patient with or without statin treatment.** (C)** Increased serum cholesterol level was linearly correlated with reduced AQP2 protein expression in the kidney specimen from patients without statins treatment (a) but not from patients with statins treatment (b). **(D)** Confocal microscopy showed increased AQP2 (green) protein expression associated with reduced ASC (red) labeling intensity after statin treatment in human kidney specimen. Cre: creatinine (µmmol/L), Chol: cholesterol (mmol/L). Original magnification, ×1000. Scale bars, 10 µm. *P < 0.05 compared with no statin treatment (Creatinine < 97 µmmol/L or Cholesterol < 5.6 mmol/L), ^#^*P* < 0.05 compared with no statin treatment (Creatinine ≥ 97 µmmol/L or Cholesterol ≥ 5.6 mmol/L).

AQP2, a key water channel located in the collecting ducts, is critically involved in the maintenance of water homeostasis in the body. In group C, immunohistochemistry (Figure 1A and B) and immunofluorescence (Figure 1D) analyses of kidney biopsy specimens showed that the density of AQP2 staining was stronger in patients with atorvastatin treatment (six samples) than those without atorvastatin (nine samples), in a manner that is independent of the levels of serum creatinine (Figure 1A and D). The diagnosis of patients with nephropathy is listed in supplementary data (S2A). To further examine the association between AQP2 protein expression and cholesterol level in serum, a correlation analysis was performed. Linear analysis study in patients without atorvastatin treatment showed a casual, negative correlation between serum cholesterol and renal AQP2 protein expression density (Figure 1Ca). A higher cholesterol level in serum is associated with a relatively low density of AQP2 protein labeling (Figure 1C*a*; *p =* 0.21, *r =* -0.43). Meanwhile, in patients with atorvastatin treatment, serum cholesterol level was found to be unrelated with AQP2 protein expression (Figure 1Cb; *p =* 0.71, *r =* 0.14).

Our recent studies 36, 37 have revealed an inhibitory role of NLRP3 inflammasome in the regulation of aquaporins in the kidneys. In order to unravel the potential mechanism by which cholesterol may affect the protein expression of AQP2, the protein expression profiles of ASC, an adaptor of NLRP3 inflammasome, were examined in the kidney specimens of patients with or without atorvastatin treatment. Confocal microscopy results shown in Figure 1D demonstrated the presence of both ASC and AQP2 in the principal cells of the collecting ducts in human kidneys. Meanwhile, in low serum cholesterol groups (Figure 1D*a* and *b*), a faint ASC staining without obvious co-localization with AQP2 was observed. Conversely, in high plasma cholesterol level groups (Figure 1D*c* and *d*), both apical ASC and AQP2 labeling were observed, which indicated good co-localization in the principal cells. Co-localization of AQP2 and ASC in Figure 1D was analyzed by calculation of the Pearson's coefficient (supplementary data S2B). Interestingly, atorvastatin treatment was found to be associated with an increased density of AQP2 staining, but a decreased density of ASC labeling (Figure 1D*b* and *d*), possibly due to a potential role of atorvastatin in blocking the activation of inflammasome, which appears to be independent of lipid-lowering.

### NLRP3 deficiency prevented downregulation of AQP2 in mice with high-fat diet

**Figure 2:**
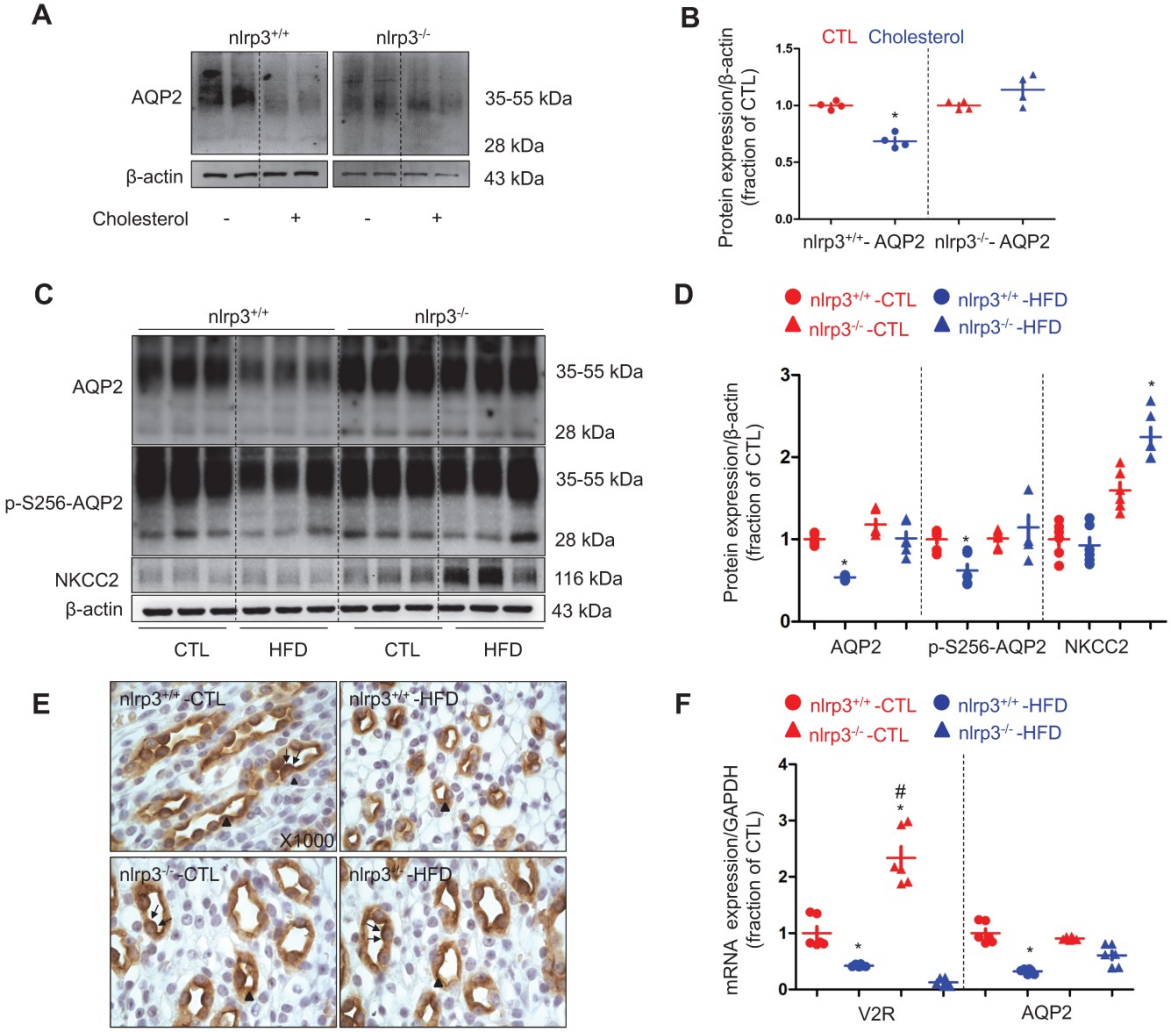
NLRP3 deficiency prevented downregulation of AQP2 in mice with high-fat diet.** (A)** and** (B)** Reduced protein expression of AQP2 after cholesterol treatment in IMCD suspensions prepared from *nlrp3*^+/+^ mice, but not in *nlrp3*^-/-^ mice. **(C)** and** (D)** Protein expression of AQP2, p-S256-AQP2, NKCC2 and corresponding semiquantitative densitometry analysis in the kidneys of *nlrp3*^+/+^ and *nlrp3*^-/-^ mice with or without HFD. **(E)** Immunohistochemistry staining of p-S256-AQP2 in the kidney sections of mice in four groups. **(F)** mRNA level of V2R and AQP2 in kidneys of *nlrp3*^+/+^ and *nlrp3*^-/-^ mice with or without HFD. Data are shown as mean ± SEM; *P < 0.05 compared with *nlrp3*^+/+^ or *nlrp3*^-/-^ CTL. ^#^*P* < 0.05 compared with *nlrp3*^+/+^ or *nlrp3*^-/-^ with HFD. CTL, control; HFD, high-fat diet, N = 5 or 6 in each group. Original magnification, ×1000. arrowheads: AQP2 expression in cytoplasm. arrows: AQP2 expression in apical membrane.

To investigate the role of NLRP3 inflammasome and its components in dyslipidemia-induced alterations of aquaporins and sodium transporters in the kidneys, a *nlrp3* gene deletion mice model was developed (supplementary data S3). The mice were fed with a high-fat diet for a duration of twelve weeks. *Nlrp3^-/-^* mice with a high-fat diet (HFD) showed a lower increment in body weight and a decreased level of visceral fat than those observed in their counterparts (Table 4, supplementary data S4). The increment in the levels of serum cholesterol and LDL in *nlrp3^-/-^* mice on a HFD was also found to be lower than that observed in *nlrp3^+/+^* mice, indicating a possible involvement of NLRP3 in the regulation of cholesterol homeostasis (Table 4). In inner medullary collecting duct cell (IMCD) suspensions prepared from *nlrp3^+/+^* mice, cholesterol significantly decreased AQP2 protein expression, in contrast, cholesterol only exhibited a minimal effect on the protein abundance of AQP2 in the *nlrp3^-/-^* mice (Figure 2A and B), indicating that NLRP3 may play a role in the modulation of cholesterol-regulated AQP2 protein level. The urine output of *nlrp3^+/+^* mice on a HFD was observed to be markedly increased by 3-folds when compared to that of *nlrp3^+/+^* controls. Meanwhile, an increased urine output (about 2-folds) was also found in *nlrp3^-/-^* mice on a HFD compared to the corresponding controls. Conversely, the urine output of *nlrp3^-/-^* mice on a HFD was observed to be lower than that of *nlrp3^+/+^* mice (Table 4). Additionally, the urinary sodium excretion was found to be increased in both mice strains on a HFD compared to that of their counterparts (Table 4). Consistently, the protein abundance and the phosphorylation of AQP2 at Serine 256 (a key phosphorylated residue for apical targeting of AQP2 to membrane, p-S256-AQP2) were observed to be markedly decreased in *nlrp3^+/+^* mice with HFD compared to those in *nlrp3^+/+^* mice without HFD; whereas the protein levels of AQP2 and p-S256-AQP2 were found to be slightly decreased in *nlrp3^-/-^* mice with HFD compared to those without HFD (Figure 2C and D). Immunohistochemistry shown in Figure 2E demonstrated that p-S256-AQP2 protein was labeled in both apical plasma membrane and cytoplasm in the principal cells of renal collecting duct in both *nlrp3^+/+^ and nlrp3^-/-^* mice. HFD diet was found to be associated with the reduced apical and intracellular labeling density of p-S256-AQP2 in *nlrp3^+/+^* mice, which was hardly detected in *nlrp3^-/-^* mice with HFD. As shown in Figure 2F, whilst *nlrp3^+/+^*mice on a HFD exhibited a significantly decreased V2R mRNA level; *nlrp3^-/-^* mice surprisingly displayed a dramatically increased V2R mRNA level compared to that of the wildtype. The V2R mRNA level could also be reduced by a HFD. In addition, HFD was observed to be associated with a decreased AQP2 mRNA level in *nlrp3^+/+^* mice, but not in *nlrp3^-/-^* mice, where the mRNA level of AQP2 was found to be mildly and insignificantly decreased (Figure 2F). In terms of renal NKCC2 protein, a Na^+^, K^+^, 2Cl^-^ cotransporter that plays an important role in the sodium reabsorption of thick ascending limbs, the protein abundance level was observed to be insignificantly lowered after a HFD in *nlrp3^+/+^* mice; conversely, *nlrp3^-/-^* mice with HFD showed a markedly increased NKCC2 protein expression (Figure 2C and D).

**Figure 3:**
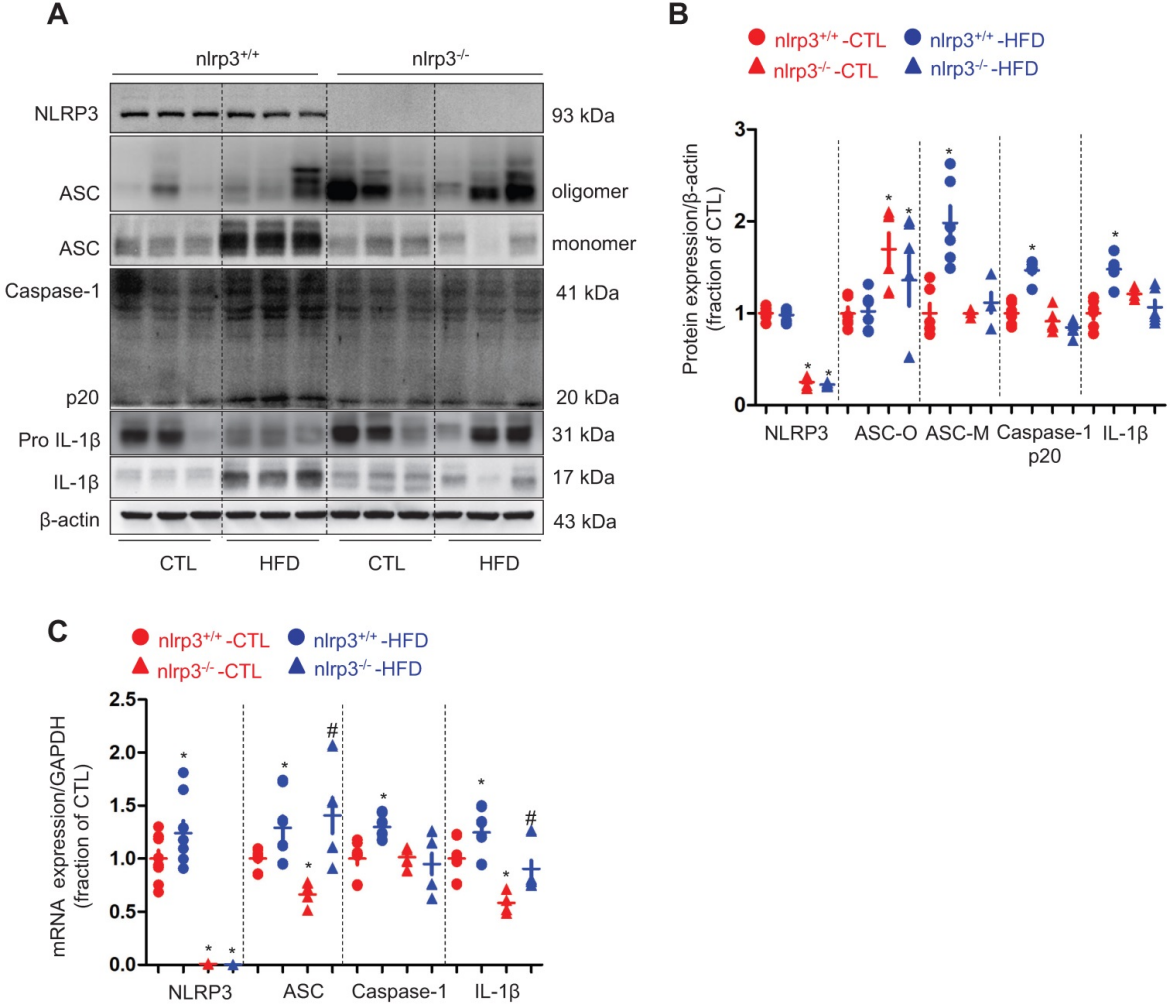
High-fat diet was associated with NLRP3 activation in the kidney of mice.** (A)** and** (B)** Protein expression of NLRP3, ASC oligomer, ASC monomer, Caspase-1 (p20), proIL-1β and IL-1β and corresponding semiquantitative densitometry analysis in the kidneys of *nlrp3*^+/+^ and *nlrp3*^-/-^ mice with or without high fat diet treatment. **(C)** mRNA level of NLRP3, ASC, Caspase-1 and IL-1β in the kidneys of *nlrp3*^+/+^ and *nlrp3*^-/-^ mice with or without HFD. Data are shown as mean ± SEM; *P < 0.05 compared with *nlrp3*^+/+^ or *nlrp3*^-/-^ CTL. ^#^*P* < 0.05 compared with *nlrp3*^+/+^ or *nlrp3*^-/-^ with HFD. CTL, control; HFD, high-fat diet, N = 5 or 6 in each group.

Next we examined a potential mechanism by which cholesterol may impact the protein expression of AQP2. Our recent studies 36, 37 have revealed an inhibitory role of NLRP3 inflammasome in the regulation of aquaporins of the kidneys. As expected, the protein expression of ASC monomer, cleaved Caspase-1 (p20) and cleaved IL-1β in the kidneys was found to be upregulated in wild type mice on a HFD; while in *nlrp3^-/-^* mice, the HFD seemed to have failed to induce significant changes in the protein expression of ASC, Caspase-1 (p20) and cleaved IL-1β expression (Figure 3A and B). Compared to *nlrp3^+/+^*mice, *nlrp3^-/-^*mice showed decreased mRNA levels of ASC and IL-1β in the kidneys (Figure 3C). In *nlrp3^+/+^* mice, the mRNA levels of NLRP3, ASC, Caspase-1 and IL-1β were observed to be markedly upregulated after a HFD; whereas in *nlrp3^-/-^* mice, the mRNA expression levels of ASC and IL-1β were also found to be increased in the kidneys after a HFD (Figure 3C).

Taken together, these findings likely indicated a potential association between NLRP3 inflammatory pathway activation and water handling in cholesterol-overloaded kidneys.

### Atorvastatin prevented downregulation of AQP2 through inhibition of ASC in the kidneys of 5/6Nx rats fed with a high-fat diet

Since dyslipidemia is common in CKD, next we further investigated whether atorvastatin can prevent abnormal regulation of water and sodium regulation in CKD with dyslipidemia. In 5/6Nx rats fed with a HFD, whilst the levels of serum cholesterol were found to be increased; whereas the clearance of creatinine was observed to be markedly decreased in about half of 5/6Nx rats, indicating a decreased glomerular filtration (Table 5). Conversely, the urine output and urinary sodium excretion, on the other hand, were found to be markedly increased following HFD treatment, suggesting a decreased tubular reabsorption of sodium and water (Table 5). As expected, atorvastatin treatment reversed the elevated levels of serum cholesterol. Interestingly, whilst atorvastatin mildly to moderately decreased the urine output and sodium excretion; it had no effect on the clearance of creatinine (Table 5).

**Figure 4:**
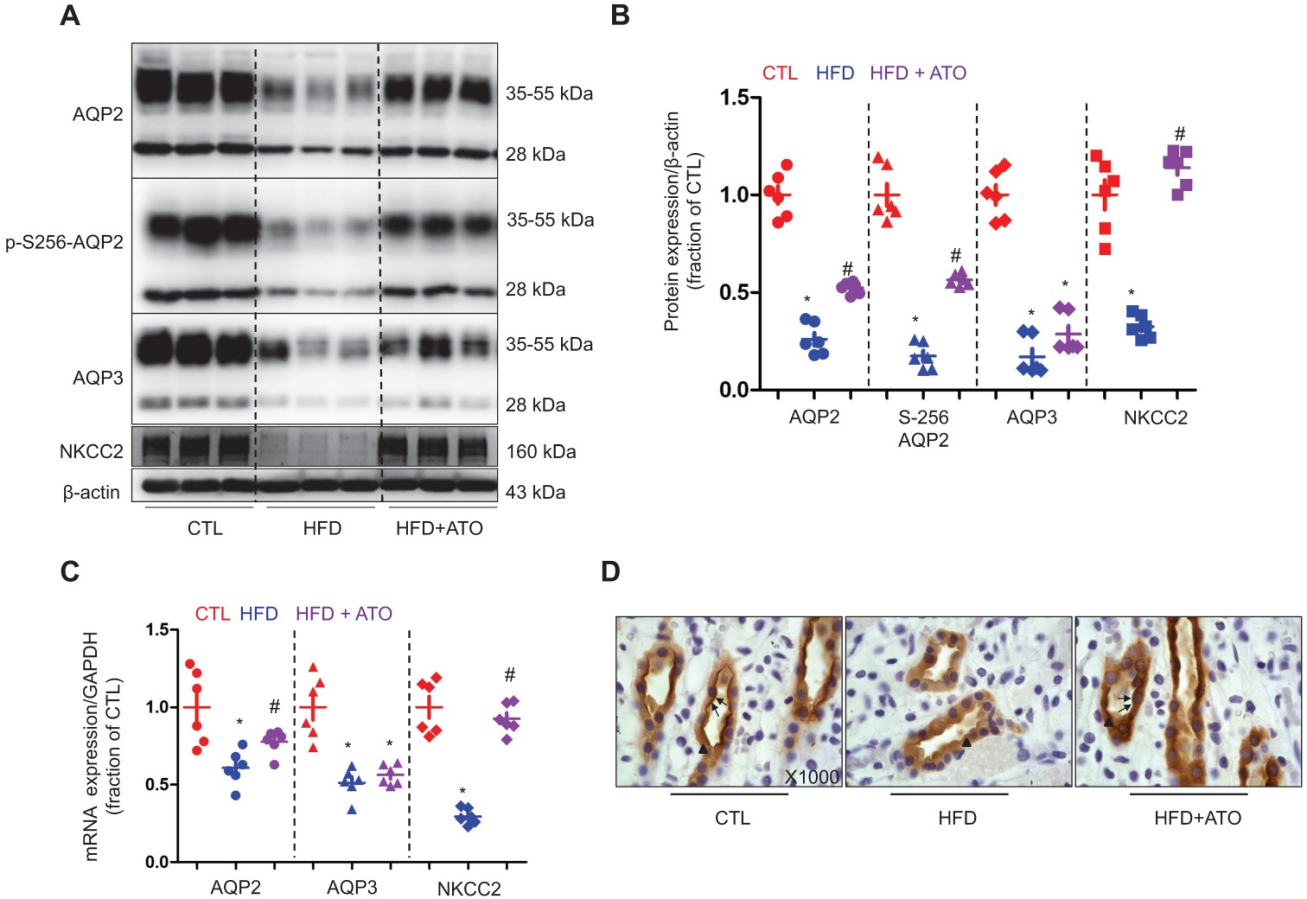
Atorvastatin prevented reduced protein expression of aquaporin and NKCC2 in the kidney of rats with 5/6Nx and high-fat diet. **(A)** and** (B)** Protein expression of AQP2, p-Ser256 AQP2, AQP3 and NKCC2 and corresponding semiquantitative densitometry analysis in the kidney of rats from three groups. **(C)** mRNA level of AQP2, AQP3 and NKCC2 in the kidney of rats from three groups. **(D)** Immunohistochemistry staining of AQP2 in the kidney sections of rats in three groups. CTL, control; HFD, high-fat diet, HFD+ATO, high-fat diet and atorvastatin, N = 6 in each group. Original magnification, ×1000. arrowheads: AQP2 expression in cytoplasm. arrows: AQP2 expression in apical membrane.

As shown in Figure 4A and B, the protein abundance levels of AQP2, p-S256-AQP2 and AQP3 were found to be dramatically decreased in the kidneys of 5/6Nx rats fed with a HFD. Immunohistochemistry results shown in Figure 4D further demonstrated that both apical and intracellular expression of AQP2 were decreased in principal cells of the medullary collecting ducts in 5/6Nx rats on a HFD. Treatment with atorvastatin improved the protein expression of AQP2, p-S256-AQP2 and AQP3 by about 1-fold, 1.2-fold and 0.5-fold, respectively in 5/6Nx rats on a HFD (Figure 4A and B). Atorvastatin was also found to be associated with an increased level of apical labeling of AQP2 in 5/6Nx rats with a HFD compared to that without a HFD. In addition, NKCC2 was observed to be dramatically downregulated in the kidneys of 5/6Nx rats with a HFD (Figure 4A and B). Again, downregulation of NKCC2 was reversed by atorvastatin treatment (Figure 4A and B). Presumably, atorvastatin-induced higher protein expression of AQPs and NKCC2 facilitate water and sodium reabsorption in 5/6Nx rats fed with a HFD. As shown in Figure 4C, the mRNA levels of AQP2 and NKCC2 were observed to be markedly decreased in the kidneys of 5/6Nx rats with a HFD, which was partially abolished by atorvastatin treatment; however, atorvastatin treatment was unable to prevent the downregulation of AQP3 mRNA expression.

**Figure 5:**
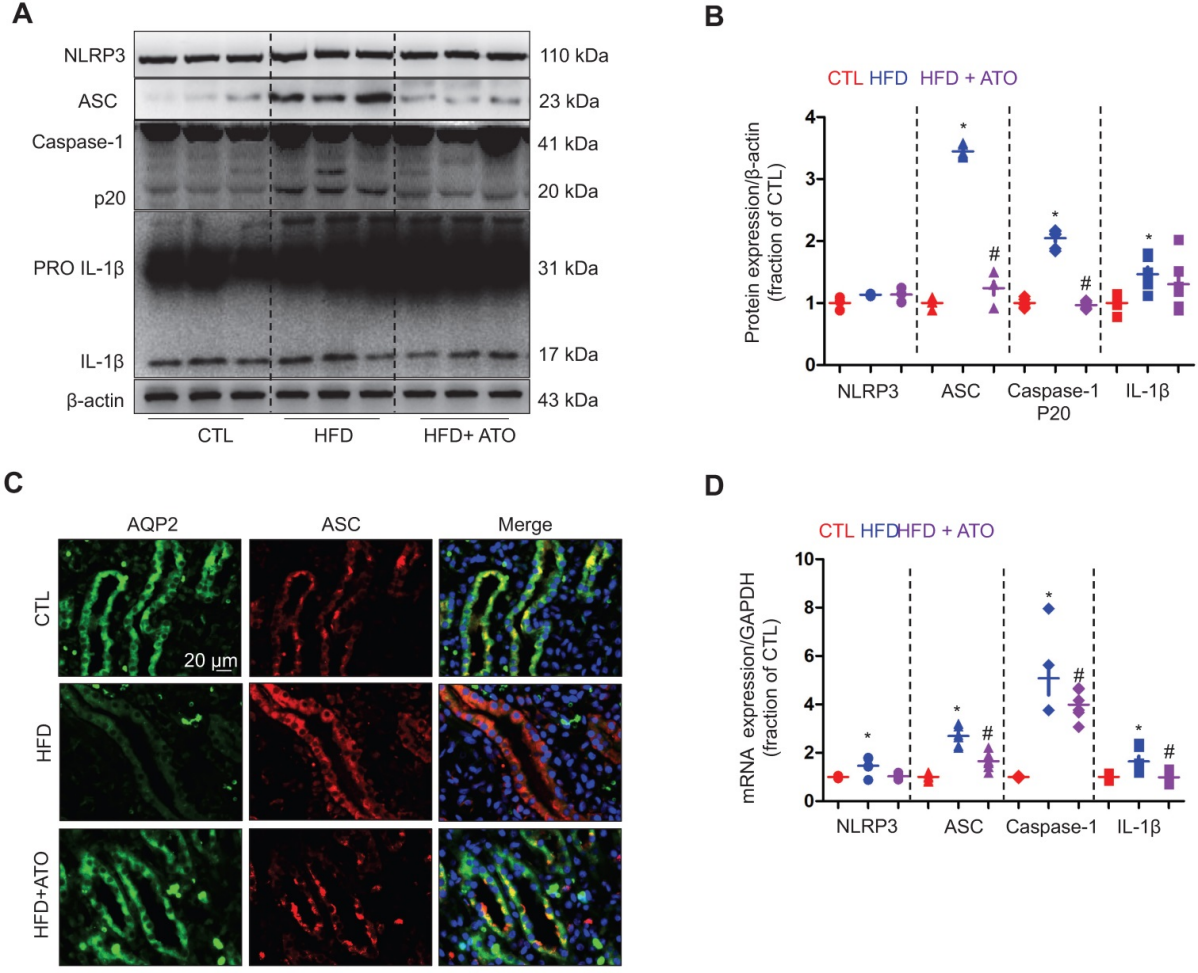
Atorvastatin inhibited increased ASC protein expression in the kidney of rats with 5/6Nx and high-fat diet.** (A)** and** (B)** Protein expression of NLRP3 inflammasome components NLRP3, ASC, Caspase-1 (P20), IL-1β and corresponding semiquantitative densitometry analysis in the kidney of rats in three groups. **(C)** Immunofluorescence showed co-localization of ASC (red) and AQP2 (green) in the collecting duct principal cells, ATO prevented reduced AQP2 and increased ASC labeling intensity in kidney inner medulla of rats from three groups. **(D)** mRNA level of NLRP3, ASC, Caspase-1 and IL-1β in the kidney of rats from three groups. Data are shown as mean ± SEM; *P < 0.05 compared with CTL. ^#^*P* < 0.05 compared with HFD. CTL, control; HFD, high-fat diet, HFD+ATO, high-fat diet and atorvastatin, N = 6 in each group. Scale bars, 20 µm.

In contrast to that observed in AQP2, whilst the protein expression levels of IL-1β were found to be higher, the protein expression levels of ASC monomer and cleaved Caspase-1 (p20) were observed to be dramatically increased in the kidneys of 5/6Nx rats with a HFD; however, the observed effects were suppressed by treatment with atorvastatin (Figure 5A and B). Immunofluorescence results as shown in Figure 5C demonstrated that AQP2 and ASC co-localizate in the principal cells of renal collecting ducts in rats. The staining density of ASC was observed to be much stronger in 5/6Nx rats with a HFD than those without a HFD. Again, the increased level of ASC labeling was attenuated by atorvastatin treatment (Figure 5C). Quantitative analysis of immunofluorescence intensity of AQP2 and ASC is shown in (supplementary data S5). As shown in Figure 5D, whilst HFD was found to be associated with increased mRNA levels of NLRP3, ASC, Caspase-1 and IL-1β; the observed effects were markedly abolished by atorvastatin treatment. These findings indicate an antagonistic role of atorvastatin in suppressing the activation of NLRP3 inflammasome.

### Atorvastatin suppressed the formation of cholesterol-induced ASC speck via the enhancement of autophagy *in vitro*

**Figure 6:**
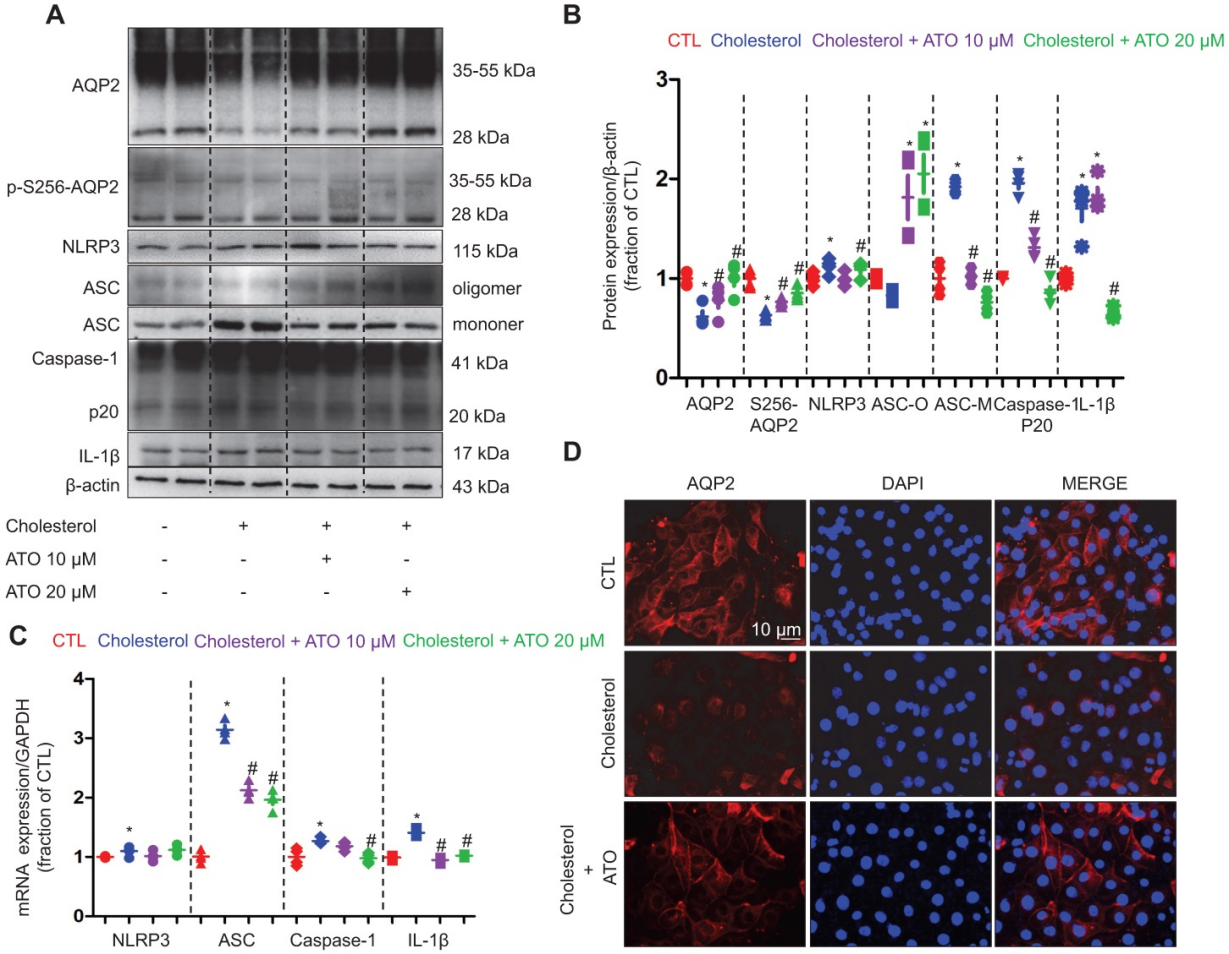
Atorvastatin prevented cholesterol-induced decrease of AQP2 protein abundance and increase of ASC monomer and IL-1β abundance in primary IMCD suspensions. **(A)** and** (B)** Protein expression of AQP2, p-Ser256 AQP2, NLRP3, Caspase-1 (p20), ASC (oligomer and monomer), IL-1β and corresponding semiquantitative densitometry analysis in cholesterol-treated primary IMCD suspensions with or without atorvastatin. **(C)** mRNA level of NLRP3, ASC, Caspase-1 and IL-1β in cholesterol-treated primary IMCD suspensions with or without atorvastatin. **(D)** Immunofluorescence of apical AQP2 in cholesterol-treated primary IMCD suspensions with or without atorvastatin. Data are shown as mean ± SEM; *P < 0.05 compared with CTL. ^#^*P* < 0.05 compared with cholesterol. CTL, control; ATO, atorvastatin. Scale bars, 10 µm.

To further examine the underlying molecular mechanism of cholesterol that causes the downregulation of AQP2 protein expression and how statins regulate the entire process, the IMCD suspension samples of rats treated with cholesterol and atorvastatin were analyzed. As shown in Figure 6A and B, treatment with cholesterol dramatically decreased the protein expression levels of AQP2 and p-S256-AQP2 compared with those in controls; however, the observed effects were markedly abolished by the pretreatment of atorvastatin at 10 and 20 μM. Immunofluorescence analysis demonstrated that, whilst AQP2 staining was observed to be dispersed throughout the cytoplasm and on the membranes; the signal was markedly decreased following cholesterol treatment in primary cultured IMCD cells. Meanwhile, treatment with atorvastatin reversed the decreased the cholesterol-induced staining intensity of AQP2 in the plasma membranes and certain intracellular compartments (Figure 6D). To examine whether V2R signaling was affected by cholesterol, dDAVP, a V2R agonist, was used to induce AQP2 protein expression in IMCD cells. As shown in supplementary Figure S6A, dDAVP (10^-7^ M) increased the protein expression level of AQP2 by two-folds in IMCD cells treated with or without atorvastatin, indicating that the AVP-V2R-AQP2 pathway remained intact in the presence of cholesterol *in vitro*. Compared to that observed with dDAVP treatment, atorvastatin (20 μM) was able to upregulate the protein expression of AQP2 by only 1.2-folds (supplementary data S6B).

As shown in Figure 6A and B, whilst the protein expression of NLRP3, ASC monomer, Caspase-1 (p20) and IL-1β was found to be markedly increased in IMCD suspension after 6 h of cholesterol treatment, the observed effects were clearly reversed by atorvastatin (20 μM). Similarly, the remarkably increased mRNA levels of ASC, Caspase-1 and IL-1β that were induced by cholesterol treatment were also found to be abolished by atorvastatin treatment (Figure 6C). These data suggest that cholesterol may activate NLRP3 inflammasome and induce the production of IL-1β in IMCD cells, which may be involved in the downregulation of AQP2 protein expression. Atorvastatin may suppress the activation of NLRP3 inflammasome at certain steps of the NLRP3 cascade.

**Figure 7:**
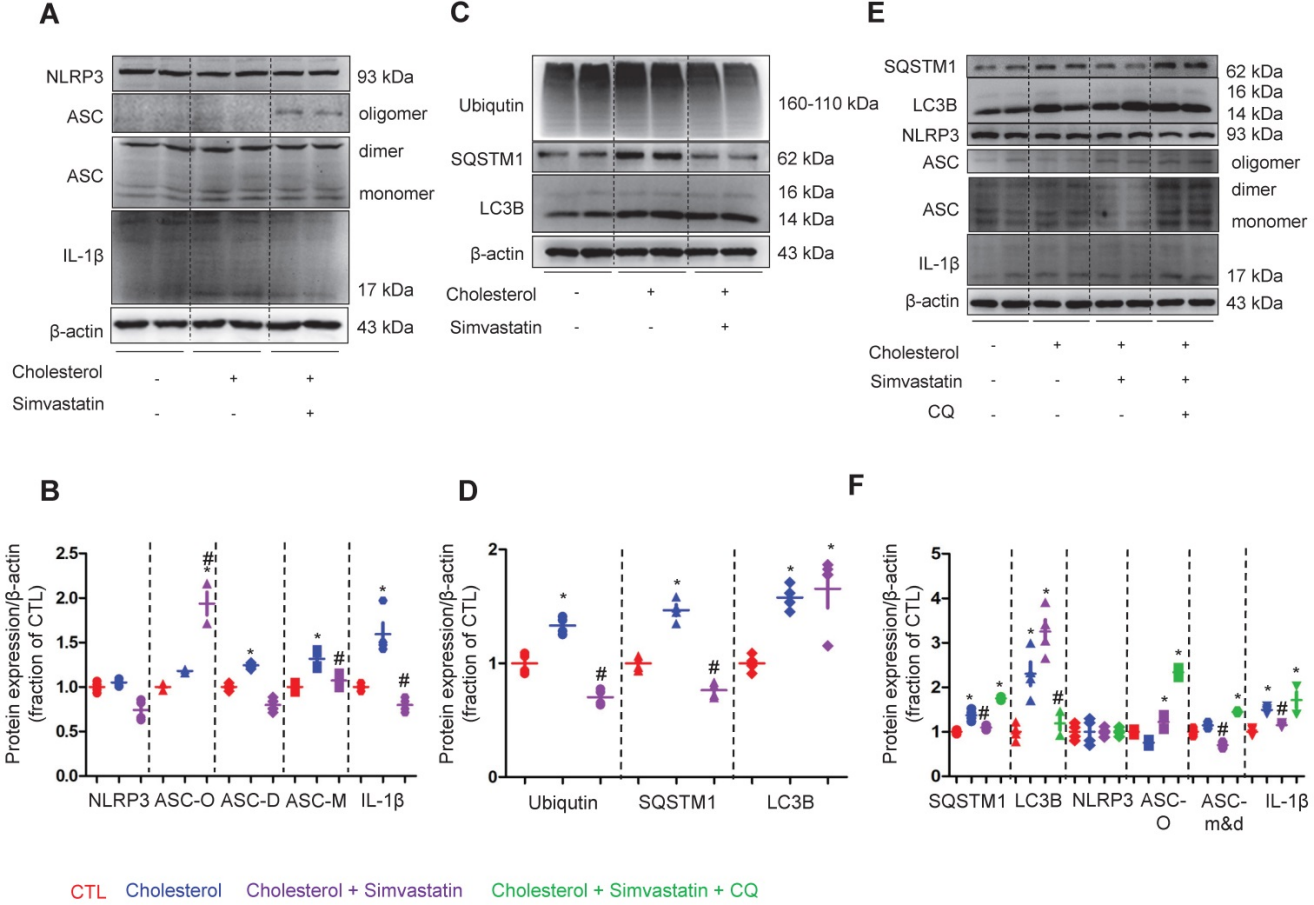
Simvastatin inhibited cholesterol-induced activation of NLRP3 inflammasome by improving autophagy stagnation in mpkCCD cells. **(A)** and **(B)** Protein expression of NLRP3, ASC (monomer, dimer and oligomer), IL-1β and corresponding semiquantitative densitometry analysis in cholesterol-treated mpkCCD cells with or without simvastatin. Simvastatin prevented cholesterol-induced decrease of AQP2 protein abundance and increase of ASC monomer and IL-1β abundance. **(C)** and** (D)** Protein abundance of ubiqutin, P62 and LC3B were detected by western blotting and corresponding semiquantitative densitometry analysis in cholesterol-treated mpkCCD cells with or without simvastatin treatment. **(E)** and** (F)** Autophagy inhibitor chloroquine (CQ) reversed inhibition of simvastatin on NLRP3 inflammasome by inducing autophagy stagnation. Protein abundance of autophagy markers and NLRP3 inflammasome components was examined by western blotting in cholesterol-treated mpkCCD cells with simvastatin and CQ. Data are shown as mean ± SEM; *P < 0.05 compared with CTL. ^#^*P* < 0.05 compared with cholesterol. CQ, chloroquine Scale bars, 10 µm.

To examine how NLRP3 inflammasome activation is suppressed by statin, the protein expression of NLRP3 inflammasome components, as well as autophagy markers were analyzed by western blot in cells of the collecting duct cell line mpkCCD. As shown in Figure 7A and B, whilst treatment with simvastatin clearly attenuated the cholesterol-induced high protein expression levels of ASC monomer and dimer in mpkCCD cells; surprisingly, simvastatin treatment markedly increased the protein expression levels of ASC oligomer that mainly play a role in the maturation and production of proinflammatory factors IL-1β 41, 42. Indeed, IL-1β protein expression was found to be downregulated by simvastatin in mpkCCD cells treated with cholesterol (Figure 7A and B).

The protein expression of ubiquitin, SQSTM1 and LC3B was observed to be increased following treatment with cholesterol. Interestingly, upon treatment with simvastatin, whilst the cholesterol-induced high protein expression of ubiquitin and SQSTM1 was almost completely abolished, the protein level of LC3B was unaffected (Figure 7C and D). Similar findings were obtained from the IMCD suspensions, where cholesterol treatment markedly induced the protein expression of SQSTM1 and LC3B, but only SQSTM1 protein expression was inhibited by atorvastatin (supplementary data S7). Next, to identify whether autophagy was involved in the statin-induced inhibition of NLRP3 inflammasome, chloroquine (CQ), an inhibitor of autophagolysosome formation was used. In mpkCCD cells treated with cholesterol, treatment with CQ fully reversed the simvastatin-induced lower protein expression of SQSTM1, which was associated with the increased protein expression of ASC dimer, monomer and IL-1β (Figure 7E and F). These data suggest a correlation between ASC and autophagy-associated degradation, which may be enhanced by statins.

**Figure 8:**
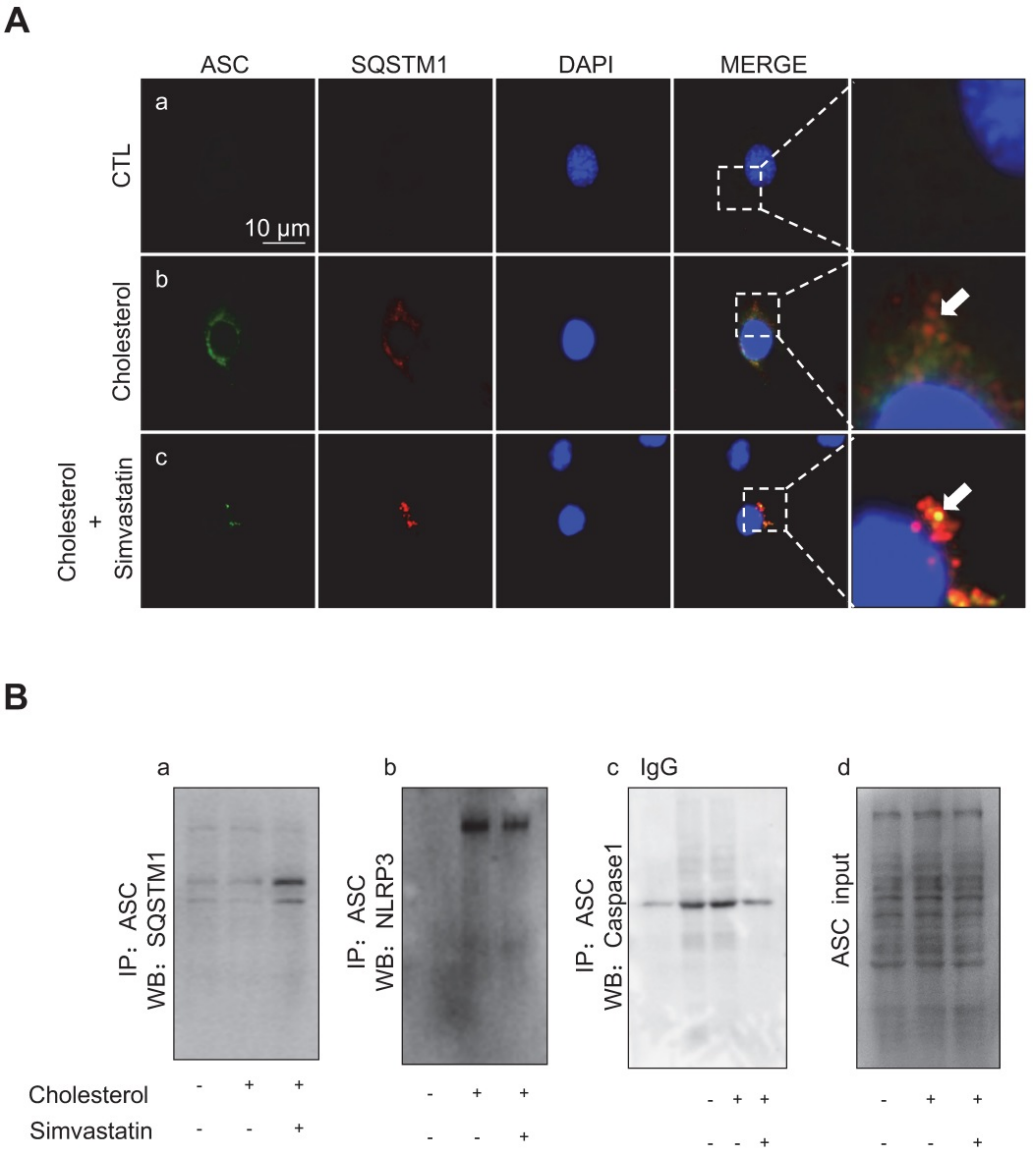
Simvastatin promoted interaction between ASC and SQSTM1 in mpkCCD cells treated with cholesterol. **(A)** Confocal microscopy showed co-expression (yellow) of ASC (green) and SQSTM1 (red) after simvastatin treatment in cholesterol-treated mpkCCD cells. Arrows indicated that ASC speck and ASC-SQSTM1 interaction in simvastatin treatment group. **(B)** Immunoprecipitation assay showed an enhanced interaction of ASC and SQSTM1 (a) induced by statin, but weak interactions of ASC with either NLRP3 (b) or Caspase-1 (c) in cholesterol-treated mpkCCD cells, (d) positive controls.

Immunofluorescence analysis showed that, whilst cholesterol treatment in mpkCCD cells caused an increased protein expression of ASC (green) and SQSTM1 (red), there was minimal co-expression of ASC and SQSTM1, indicating that the ASC speck formation was scarce (Figure 8A). Conversely, simvastatin treatment-induced clear co-expression of ASC and SQSTM1, indicating a markedly increased formation of the ASC speck (Figure 8A). Co-immunoprecipitation (IP) further confirmed that ASC was physically associated with SQSTM1, and that simvastatin indeed strengthened such an association. As shown in Figure 8B*a*, ASC exhibited an interaction with SQSTM1 in mpkCCD cells, and the ASC-SQSTM1 interaction was dramatically increased in the presence of simvastatin. Meanwhile, the cholesterol-induced strong interactions of ASC-NLRP3 (Figure 8B*b*) and ASC-Caspase-1 (Figure 8B*c*) were both observed to be markedly inhibited by simvastatin treatment. These data suggest that cholesterol accumulation can induce the expression of NLRP3 inflammasome components in mpkCCD cells. Simvastatin, through enhancing autophagy can promote ASC clearance and thus inhibit the activation of NLRP3 inflammasome.

## Discussion

Obesity-related renal alterations, such as hemodynamic changes, impaired natriuresis, and activated renal (and/or systemic) renin-angiotensin-aldosterone system, have been presumed to contribute to the maintenance or development of hypertension 30. The current study demonstrated that an increased cholesterol level may promote excretion of water and sodium through the induction of NLRP3 inflammasome activation, which then decreases the protein expression of tubular aquaporins and sodium transporters in the kidneys. Results from this study also showed that, statins, through the improvement of autophagy flux and the promotion of ASC clearance, can inhibit the activation of NLRP3 inflammasome, and thus upregulate the expression of the protein components in a manner that is independent of the lipid-lowering effects.

An early prospective cohort study has demonstrated the significant associations between the abnormal cholesterol parameters and both the elevated creatinine and reduced GFR in a follow-up with a duration of 14 years in average 4, indicating lipid-lowering drugs are necessary in order to prolong the normal renal function or to prevent renal dysfunction. However, it has been shown that statins may be unable to lower the incidence of ESRD or to induce a decline in eGFR 19 and have little or no effect in patients receiving dialysis 18. At some point of treatment, it has been shown that statins may increase the risk of developing severe renal failure 20. Our retrospective cohort study showed casual positive correlations between total cholesterol levels and both urine volume and sodium excretion, which was associated with slightly decreased eGFR. Atorvastatin was observed to lower serum cholesterol and LDL, without affecting eGFR when eGFR > 60ml. However, when eGFR < 60, statin insignificantly and mildly decrease eGFR compared to that with the corresponding controls. This is in agreement with the observation that atorvastatin treatment had no effect on the improvement of eGFR despite the lowered level of lipid. AVP-regulated AQP2 is a key aquaporin in the collecting ducts that determines the final urine output. The abundance of AQP2 protein expression was found to be negatively linear correlated with serum cholesterol levels. Our results also showed that statin treatment increased the AQP2 expression in the kidney specimen and abolished the linear correlation between AQP2 expression and serum cholesterol levels in patients, indicating that statins may play a role in the regulation of AQP2 protein expression.

Data from our animal studies are consistent with the findings from kidney biopsy. In mice on a HFD, the increased urine output was found to be associated with decreased protein and mRNA expression of AQP2. Interestingly, the mRNA levels of V2R were observed to be downregulated in the kidneys of mice on a HFD, indicating that a high fat may impair the V2R-AQP2 regulatory pathway; however, the activity of V2R seemed to be unaffected, as dDAVP was observed to be capable of upregulating the protein expression of AQP2 in IMCD cells treated with cholesterol. Evidence has shown that people with obesity exhibit an increased level of vasopressin 26, 27 and that increased plasma AVP is associated with insulin resistance, adiposity and metabolic syndrome 28. Therefore, a decreased mRNA level of V2R may, to a certain extent, attenuate AVP-mediated water and sodium reabsorption in the kidneys, and therefore avoid water retention in obesity, especially during low renal filtration. In 5/6Nx rats on a HFD, the renal filtration ability was observed to be impaired in-line with a decreased Ccr. Although treatment with atorvastatin was unable to restore the level of Ccr, there were markedly lowered levels of urine output and sodium excretion, possibly through the increased protein expression of AQP2 and NKCC2.

Increased generation of inflammatory cytokines induced by lipid accumulation that causes structural and functional changes in glomerular and tubular cells has been regarded as one of the potential mechanisms in progressive renal injury during dyslipidemia 30. Cholesterol-induced inflammation can at least partially mediate the downregulation of heightened protein expression. Our *in vitro* study using IMCD cells showed that cholesterol treatment markedly induced the low protein expression of AQP2, which was associated with the activation of NLRP3 inflammasome. Particularly, the protein expression of adaptor ASC monomer that is critical for maturation of IL-1β was found to be increased. Our previous studies have demonstrated that IL-1β can decrease gene and protein expression of AQP2 in the principal cells of collecting ducts 35-37.

Our data showed that in the early stages of dyslipidemia, the kidneys may decrease the reabsorption of water and sodium by downregulating the water channels and sodium cotransporters, and thus potentially attenuate the water and sodium retention, and finally delay the development of hypertension. In addition, when GFR was lowered due to the advancement of nephropathy in obesity, the tubular reabsorption of sodium and water in these sites should be decreased as well due to glomerulotubular balance. Therefore, the observed elevated level of inflammatory cytokine IL-1β and the decreased level of transporters/aquaporins may be beneficial to dyslipidemia-induced renal alterations. Our data suggest that statins may enhance water and sodium reabsorption in the kidneys of rats with a HFD, likely through the upregulation of AQP2 and NKCC2 protein expression. However, the effect of statins on water and sodium metabolism is probably not favorable in patients and animals with dyslipidemia, especially when the renal function is compromised. A lower excretion of water and sodium may increase the extracellular volume and thus lead to the elevation of blood pressure. Presumably, persistent, impaired natriuresis and diuresis after statin treatment may contribute to the maintenance and development of hypertension, one of the most common comorbidities associated with obesity.

Some studies have demonstrated that statins can increase the expression of AQP2 in the principal cells of collecting ducts. Statins have been shown to stimulate the expression of AQP2 and NKCC2 by a vasopressin-independent mechanism that leads to water retention, decreased urine volume, and increased urine osmolality in animals 24.* In vitro* experiments have shown that statins can increase the apical membrane expression of AQP2 by lowering the plasma membrane cholesterol 23, 25. Conversely, this study demonstrated that, through the inhibition of cholesterol-induced inflammation, statins can increase the protein expression of AQP2 and NKCC2, which may potentially promote water and sodium retention. Meanwhile, our data also showed that, in a manner that is independent of lowered cholesterol, statins can exert a direct effect on inflammation by suppressing the function of ASC oligomer and thus decreasing the cholesterol-induced maturation and production of inflammatory cytokine IL-1β.

NLRP3 inflammasome is the central hub of the inflammatory response system that elicits a pro-inflammatory response. Upon activation, the NLRP3 sensor oligomerization typically nucleates the polymerization of ASC into highly cross-linked macromolecular assemblies as ASC specks, which act as a molecular bridge between danger-signal sensors (NLRP3 in the context of this study) and procaspase-1 41. By auto-activation, caspase-1 subsequently cleaves pro-IL-1β/pro-IL-18 into the corresponding matured form IL-1β/IL-18, which is then released into the extracellular space by exocytosis or loss of membrane integrity 43, causing inflammatory responses and pathological effects. The recruitment and self-assembly processes of the ASC is one of the key steps in the activation of NLRP3 inflammasome. The expression and the oligomeric states of ASC are tightly regulated to prevent aberrant inflammatory responses 41, 42. In cholesterol-overloaded mpkCCD cells, the markedly increased protein expression of ASC (monomer and dimer) and IL-1β were abolished by statin treatment as expected, although statin treatment was found to be associated with the formation of ASC specks. Since the formation of ASC specks has been considered as a marker and signal of inflammatory activation 44, the significance of statin-induced ASC specks was further investigated.

**Figure 9:**
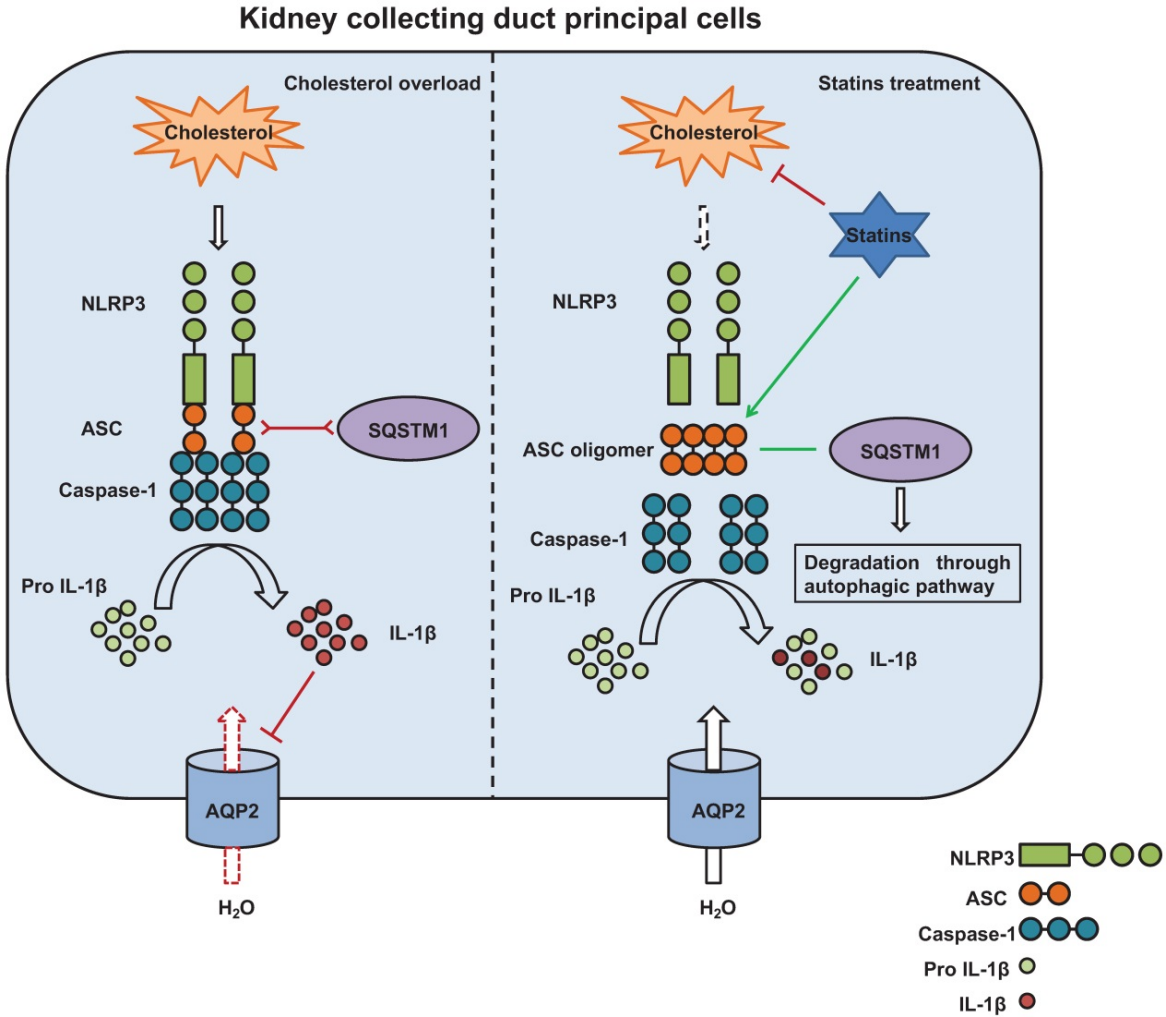
The potential mechanism by which statins inhibit activation of NLRP3 inflammasome induced by cholesterol and promotes protein expression of aquaporins. Cholesterol stimulates assembly of NLRP3 inflammasome complex and activation of Caspase-1. Caspase-1 cleaves pro-IL-1β to its biologically active forms IL-1β which downregulates AQP2 protein expression (left). Statins, on one hand, by inhibiting synthesis of cholesterol and decreasing cholesterol levels, attenuate production of IL-1β; on the other hand, statins promote formation of ASC speck and the interaction between ASC and SQSTM1, leading to degradation of ASC by autophagy. By this way, statins inhibit activation of Caspase-1 and maturation of IL-1β, maintaining AQP2 protein expression (right).

Autophagy has emerged as one of the essential processes in the modulation of excess inflammasome activation 45. Interestingly, our results showed that cholesterol treatment can induce an increased expression of SQSTM1 that co-expressed with ASC without overlapping in mpkCCD cells, and that statin treatment can promote co-localization of ASC and SQSTM1, indicating an interaction between ASC and autophagy marker SQSTM1. In contrast, co-IP assays showed a decreased co-expression of ASC with either NLRP3 or caspase-1 after statin treatment compared with that with cholesterol treatment alone. These data indicate that ASC specks may probably be cleared through autophagy, especially in the presence of statin. In agreement with this, a bioinformatics study has shown that both SQSTM1 or caspase-1 can interact with the same QYQA domain of ASC 46. To verify the hypothesis, chloroquine CQ, an inhibitor of autophagolysosome, was used to induce autophagy stagnation in mpkCCD cells treated with cholesterol and statin. Our results revealed that CQ can effectively reverse the statin-induced decreased protein expression of SQSTM1, and increase the protein expression of ASC (monomer, dimer and oligomer) and IL-1β, suggesting that statin may promote autophagy flux and improve autophagy stagnation 47 induced by overloaded-cholesterol in mpkCCD cells. Through interaction with SQSTM1, ASC specks may be cleared by autophagy and degradation, which are possibly enhanced by statin. Thus, statin can suppress cholesterol-induced inflammatory response (and IL-1β production) and improve the expression of several target proteins, such as AQP2 (Figure 9). Taken together, the data suggest that statin can induce the oligomerization of ASC and lead to SQSTM1-dependent selective autophagy, thus attenuate inflammation events associated with NLRP3 inflammasome and ultimately recover the expression of target proteins involved in regulation of water and sodium in the kidneys.

There may be some other mechanisms by which statins regulate cholesterol-associated activation of NLRP3 inflammasome. Activation of the NLRP3 inflammasome is a two-step process requiring a priming step (signal 1) followed by an activation step (signal 2). Signal 1 stimulates the production of pro-IL-1β. Although our mechanistic study revealed that autophagy linked protein degradation played the key role in modulating the NLRP3 inflammasome stability, it should be noted that ASC and IL-1β gene expression was upregulated by cholesterol and inhibited by statin, suggesting that NLRP3 inflammasome priming was initially regulated, and statin or cholesterol may directly regulate the transcriptional activity of ASC and IL-1β.

Cholesterol may also stimulate signal 2. Recent studies have documented an important role of the NLRP3 inflammasome in crystalline nephropathy, showing that crystals may harm tubular cells by activating NLRP3 inflammasome 48. Marked increases of renal phospolipid and cholesterol levels and intratubular lipid droplets after a Western diet have been reported recently 31 49. Crystalline cholesterol, acting as an endogenous danger signal, has an agonistic potential on NLRP3 and trigger IL-1β secretion 50. This process contributes to cholesterol-induced tubular inflammation and necroptosis 51, also, decrease of AQP2 expression as seen in the current study. Statins decreased volume expansion of cholesterol crystals by blunting sharp-tipped crystal structure and dissolving cholesterol crystals 52, thus indirectly suppressing activation of NLRP3 inflammasome and rescuing AQP2 expression. Additionally, further studies will be necessary to investigate the role of statin or cholesterol in NLRP3 priming and activation.

In conclusion, our data in retrospective clinical cohort and animal studies demonstrated that increased cholesterol levels are associated with decreased expression of renal aquaporin, which may be beneficial in the early stages of dyslipidemia or in CKD to promote the excretion of water and sodium. Statins can ameliorate cholesterol-induced inflammation through the inhibition of ASC speck formation, improve the expression of associated proteins, and potentially lead to impaired natriuresis and diuresis. Further clinical and animal studies are required to precisely clarify the role of statins in volume regulation during dyslipidemia.

## Supplementary Material

Supplementary figures and tables.

## Figures and Tables

**Table 1:** Baseline characteristics of participants by quartile of total cholesterol (Group A)

	Total cholesterol (mmol/L)		*P* value
	Low quartile	High quartile	
Range, Total cholesterol (mmol/L)	2.28, 4.67	4.68, 8.71	
N	116	115	
Age (years)	40.2 ± 8.7	41.4 ± 9.0	0.282
Sex (male %)	54 (46.6)	46 (40.0)	0.315
BMI (kg/m^2^)	23.9 ± 5.2	24.9 ± 4.1	0.105
**Serum laboratory values**			
FBG (mmol/L)	5.1 ± 1.1	5.4 ± 1.4	0.035
OGTT-2h (mmol/L)	8.2 ± 3.1	9.7 ± 3.9	0.007
Total cholesterol	4.0 ± 0.5	5.5 ± 0.7	< 0.001
LDL-C (mmol/L)	2.3 ± 0.5	3.5 ± 0.8	< 0.001
Serum sodium (mmol/L)	139.7 ± 2.4	139.8 ± 2.4	0.801
Serum potassium (mmol/L)	3.7 ± 0.4	3.7 ± 0.5	0.273
BUN (mmol/L)	4.8 ± 1.4	5.2 ± 1.4	0.049
Serum creatinine (μmol/L)	87.5 ± 18.1	88.1 ± 19.3	0.793
eGFR (ml/min·per 1.73 m^2^)	79.3 ± 14.1	76.7 ± 13.9	0.171
**Urine laboratory values**			
Urine volume (mL/ 24 h)	1894 ± 724.9	2121 ± 680.9	0.015
Urinary sodium (mmol/ 24 h)	122.2 ± 60.6	140.4 ± 61.0	0.024
Urinary potassium (mmol/ 24 h)	33.2 ± 13.4	36.1 ± 15.2	0.134
UAER (mg/ 24 h)	11.8 ± 16.2	16.0 ± 20.9	0.201

BMI, body mass index; FBG, fasting blood glucose; OGTT, oral glucose tolerance test; LDL-C, low density lipoprotein cholesterol; BUN, blood urea nitrogen; eGFR, estimated glomerular filtration rate; UAER, urinary albumin ejection rate.

**Table 2:** Associations between serum cholesterol and other biochemistry parameter (n = 231) (Group A)

Variable	Serum cholesterol, mmol/L	
	*r*	*P*
eGFR, ml/min·per 1.73 m^2^	-0.137	0.038
serum sodium, mmol/L	0.027	0.708
urinary sodium, mmol/ 24 h	0.167	0.011
urine volume, mL/ 24 h	0.178	0.007

Adjusting for age and sex.

**Table 3:** Lipid profile in patients classified by eGFR (Group B)

All cohort	TC (mmol/L)	LDL-C (mmol/L)	Hs-CRP (mmol/L)	eGFR (ml/min per 1.73 m^2^)
CTL (n = 84)	5.37 ± 0.07	3.28 ± 0.05	8.43 ± 0.70	75.04 ± 1.46
ATO (n = 50)	4.67 ± 0.15*	2.76 ± 0.11 *	3.49 ± 0.51 *	74.10 ± 2.35
a: eGFR ≥ 90 (ml/min·per 1.73 m^2^)				
	**TC (mmol/L)**	**LDL-C (mmol/L)**	**Hs-CRP (mmol/L)**	**eGFR (ml/min per 1.73 m^2^)**
CTL (n = 8)	5.36 ± 0.28	3.31 ± 0.20	6.72 ± 1.03	101.15 ± 3.31
ATO (n = 8)	4.26 ± 0.22*	2.51 ± 0.17*	1.88 ± 0.70 *	99.31 ± 3.53
b: 60 ≤ e GFR < 90 (ml/min·per 1.73 m^2^)				
	**TC (mmol/L)**	**LDL-C (mmol/L)**	**Hs-CRP (mmol/L)**	**eGFR (ml/min per 1.73 m^2^)**
CTL (n = 68)	5.36 ± 0.08	3.26 ± 0.06	7.34 ± 0.44	75.96 ± 1.26
ATO (n = 34)	4.78 ± 0.21*	2.40 ± 0.11 *	3.00 ± 0.47 *	75.66 ± 2.19
c: eGFR < 60 (ml/min·per 1.73 m^2^)				
	**TC (mmol/L)**	**LDL-C (mmol/L)**	**Hs-CRP (mmol/L)**	**eGFR (ml/min per 1.73 m^2^)**
CTL (n = 8)	5.47 ± 0.25	3.37 ± 0.20	19.47 ± 5.05	56.24 ± 1.42
ATO (n = 8)	4.60 ± 0.20*	2.74 ± 0.13 *	7.23 ± 1.97*	50.23 ± 3.12

ATO, atorvastatin; TC, total cholesterol; LDL-C, low density lipoprotein cholesterol; Hs-CRP, hypersensitive-CRP; eGFR, estimated glomerular filtration rate; * p < 0.05 when compared with CTL.

**Table 4:** Functional data of *nlrp3^+/+^* and *nlrp3*^-/-^ mice with or without HFD

Groups	*nlrp3^+^/^+^*-CTL (n = 5)	*nlrp3^+^/^+^*-HFD (n = 6)	*nlrp3^-^/^-^*-CTL (n = 6)	*nlrp3^-^/^-^*-HFD (n = 6)
Body Weight (g)	35.0 ± 1.1	48.3 ± 2.2^*^	30 ± 1.2^*^	32.7 ± 1.3^*$^
S-Chol (mmol/L)	2.57 ± 0.35	4.28 ± 0.70^*^	2.29 ± 0.04	2.48 ± 0.21^$^
S-TG (mmol/L)	0.38 ± 0.02	0.63 ± 0.04^*^	0.67 ± 0.01	0.45 ± 0.08^#$^
S-HDL (mg/dL)	1.75 ± 0.25	2.87 ± 0.49^*^	1.54 ± 0.01	1.70 ± 0.12^$^
S-LDL (mg/dL)	0.56 ± 0.07	1.03 ± 0.18^*^	0.55 ± 0.04	0.61 ± 0.10^$^
S-FFA (μmol/dL)	864 ± 77	915 ± 88^*^	742 ± 12^*^	780 ± 100^$^
S-Na^+^ (mmol/L)	156 ± 0.1	155 ± 0.7	156 ± 0.9	159 ± 0.9
S-K^+^ (mmol/L)	3.1 ± 0.1	3.4 ± 0.1	3.4 ± 0.1	3.0 ± 0.1
S-urea (mmol/L)	9.07 ± 0.44	9.85 ± 1.25	9.18 ± 0.58	12.1 ± 1.26
S-Cr (mmol/L)	10 ± 1	9 ± 0.5	11 ± 0.7	10 ± 0.3
Ccr (μl/min/g)	10 ± 1.2	9.1 ± 0.5	12 ± 0.7	10 ± 0.3
UO (ml)	0.43 ± 0.12	1.20 ± 0.16^*^	0.37 ± 0.14	0.84 ± 0.23^#$^
U-ALB (μg/24h)	3.2 ± 0.9	39.3 ± 7.6^*^	1.9 ± 1.57	11.4 ± 2.3^#$^
UACR	0.11 ± 0.01	0.68 ± 0.14^*^	0.07 ± 0.03	0.40 ± 0.04^#$^
U-Na^+^ (mmol/L)	119 ± 10	90 ± 4^*^	77 ± 15^*^	162 ± 22^#$^
U_Na_V (μmol/ 24 h)	52 ± 16	90 ± 16^*^	33 ± 16^*^	132 ± 36^#$^
FE_Na_	0.58 ± 0.03	0.38 ± 0.07^*^	0.70 ± 0.07^*^	0.67 ± 0.07^$^
Na_balance_ (μmol/ 24 h)	547 ± 36	949 ± 138^*^	538 ± 43	347 ± 31^#$^

HFD, high-fat diet; S-Chol, serum cholesterol; S-TG, serum triglycerides; S-HDL, serum high density lipoprotein; S-LDL, serum low density lipoprotein; S-FFA, serum free fatty acid; Ccr, clearance rate of endogenous creatinine; UO, urine output; U-ALB, urine albumin; UACR, urine albumin to creatinine ratio; U_Na_V, urine Na^+^volume; FE_Na,_ fractional excretion of filtrated sodium; Na_balance_, regulation of sodium balance; *p < 0.05 when compared with *nlrp3^+/+^* controls, ^#^*p* < 0.05 when compared with *nlrp3^-/-^* controls, ^$^*p* < 0.05 when compared with *nlrp3^+/+^*-HFD.

**Table 5:** Functional data in rats from SHAM, 5/6Nx+HFD, 5/6Nx+HFD+ATO

Groups	Sham (n = 6)	5/6Nx+HFD (n = 6)	5/6Nx+HFD+ATO (n = 6)
Body Weight (g)	341.5 ± 1.1	464.8 ± 2.2^*^	431.8 ± 1.2^*#^
S-Chol (mmol/L)	1.03 ± 0.18	1.59 ± 0.09^ *^	1.18 ± 0.11^#^
S-TG (mmol/L)	0.32 ± 0.02	0.39 ± 0.06	0.36 ± 0.09
S-HDL (mg/dL)	0.97 ± 0.10	1.08 ± 0.14	0.74 ± 0.24
S-LDL (mg/dL)	0.32 ± 0.03	0.52 ± 0.05^*^	0.49 ± 0.07^*^
Glu (mmol/dL)	8.50 ± 1.03	7.61 ± 0.97	8.12 ± 1.23
S-Cr (μmol/L)	29 ± 4.2	43 ± 4.1^*^	46 ± 5.2^*^
S-urea (mmol/L)	5.2 ± 0.5	16 ± 2*	13 ± 1.2^*#^
S-Hcy (mmol/L)	4.88 ± 0.85	5.09 ± 0.52	5.69 ± 0.36
UO (ml)	9.51 ± 0.75	16.18 ± 0.80^*^	10.31 ± 0.72^#^
Ccr (μl/min/g)	9.62 ± 1.2	5.40 ± 0.5^*^	4.63 ± 0.7^*^
U-Na^+^ (mmol/L)	17 ± 3	55 ± 11^*^	35 ± 8^*#^
U_Na_V (μmol/24h)	157 ± 4	445 ± 12^*^	316 ± 8^*#^

5/6Nx, 5/6 kidnectomy; HFD, high-fat diet; ATO, atorvastatin; S-Chol, serum cholesterol; S-TG, serum triglycerides; S-HDL, serum high density lipoprotein; S-LDL, serum low density lipoprotein; Glu, serum glucose; S-Cr, serum creatinine; S-Hcy, serum homocysteine; UO, urine output;Ccr, clearance rate of endogenous creatinine; U_Na_V, urine Na^+^volume; *p < 0.05 when compared with Sham, ^#^*p* < 0.05 when compared with 5/6Nx+HFD.
