# Supplementary material for: Statins ameliorate cholesterol-induced inflammation and improve AQP2 expression by inhibiting NLRP3 activation in the kidney
Source: Theranostics. 2020 Aug 20;10(23):10415–33. doi: 10.7150/thno.49603 (PMC7482822; doi:10.7150/thno.49603)

SUPPLEMENTARY DATA

S1

Primer sequences for RT-PCR (mouse)

Target Gene	Primer Sequence
NLRP3 F	ATTACCCGCCCGAGAAAGG
NLRP3 R	CATGAGTGTGGCTAGATCCAAG
Caspase-1 F	ACAAGGCACGGGACCTATG
Caspase-1 R	TCCCAGTCAGTCCTGGAAATG
ASC F	ACAGGCTGTGATACGACTAAAGA
ASC R	CATTTGCCGTTTCGGTGGA
IL-1βF	GAAATGCCACCTTTTGACAGTG
IL-1βR	TGGATGCTCTCATCAGGACAG
V2R F	GCTGTGGCTCTGTTTCAAGTG
V2R R	CCAGGATCATGTAGGAAGAGGC
AQP2 F	GGACCTGGCTGTCAATGCTC
AQP2 R	GCGGGCTGGATTCATGGAG

Primer sequences for RT-PCR (Rat)

Target Gene	Primer Sequence
AQP2 F	TGACCTCAACTACATGGTCTACA
AQP2 R	GCGGGCTGGATTCATGGAG
AQP3 F	AGCAGATCTGAGTGGGCAGT
AQP3 R	CTTGGGCTTAAGAGGGGAAC
NKCC2 F	CGGGTCGTCTAGATCCAAAA
NKCC2 R	ATGGACTTGGAAACGACTGG
NLRP3 F	CGAGACCTCTGGGAAAAAGCT
NLRP3 R	GCATACCATAGAGGAATGTGATGTACA
Caspase-1 F	GATGGCACATTTCCAGGACTGA
Caspase-1 R	TGTTGCAGATAATGAGGGCAAGAC
ASC F	ACTTCTGTGACCCTGGCAATGAG
ASC R	GCTGAGCAGCTGCAAACGAC
IL-1βF	GAAATGCCACCTTTTGACAGTG
IL-1βR	TGGATGCTCTCATCAGGACAG

S2A

Diagnosis of patients with kidney diseases in Figure1

Diagnosis	Case Number
IgA nephropathy	2006, 2007, 2018, 2027, 2035, 2036
Nephrotic syndrome	2013, 2016, 2028, 2029, 2037, 2039
Interstitial nephritis	2024
ANCA related vasculitis/CKD stage 5	2008
Lupus nephritis	2025
Chronic glomerulonephritis	2043
Henoch-Schonlein purpura	2011

Supplementary data figure legends:

S2B Figure. Co-localization of AQP2 and ASC in Figure 1D was analyzed by calculation of the Pearson's coefficient.

S3 Figure. Genotyping of *nlrp3*$^{+/+}$ and *nlrp*3$^{-/-}$ mice

S4 Figure. Increased body size and abdominal fat in *nlrp3*$^{+/+}$ mice compared to *nlrp3*$^{-/-}$ mice with HFD for 12 weeks.

S5 Figure. Quantitative analysis of immunofluorescence intensity of AQP2 and ASC in Figure 5C.

S6 Figure. (A) Protein expression of AQP2 in cholesterol-treated primary IMCD suspensions with or without dDAVP 10^{-7} M treatment. **(B)** Protein expression of AQP2 in primary IMCD suspensions with or without ATO treatment.

S7 Figure. (A) and **(B)** Protein expression of SQSTM1 and LC3B in

cholesterol-treated primary IMCD suspensions with or without ATO treatment.

Supplementary data: S2B

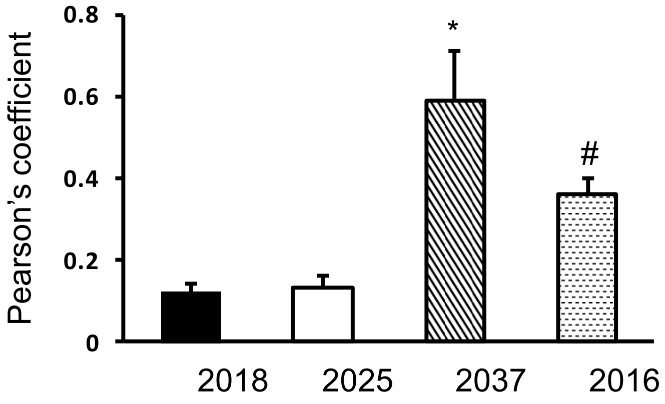

Supplementary data: S3

A

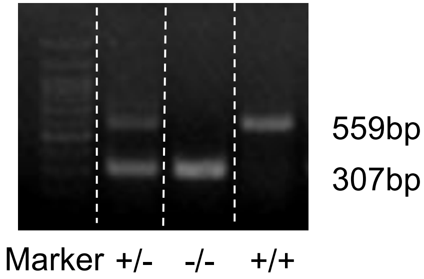

Supplementary data: S4

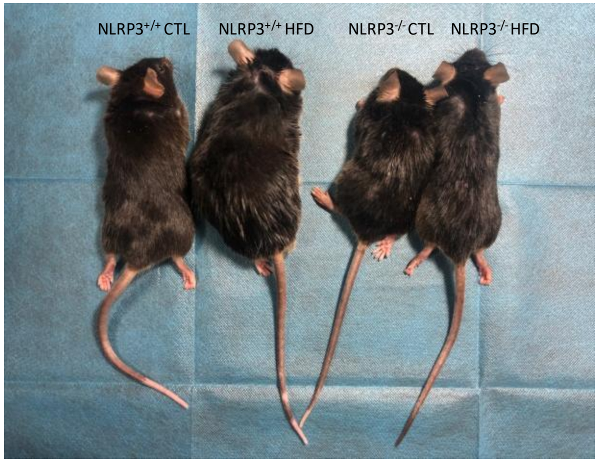

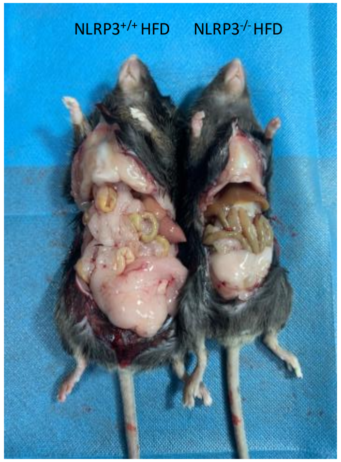

Supplementary data: S5

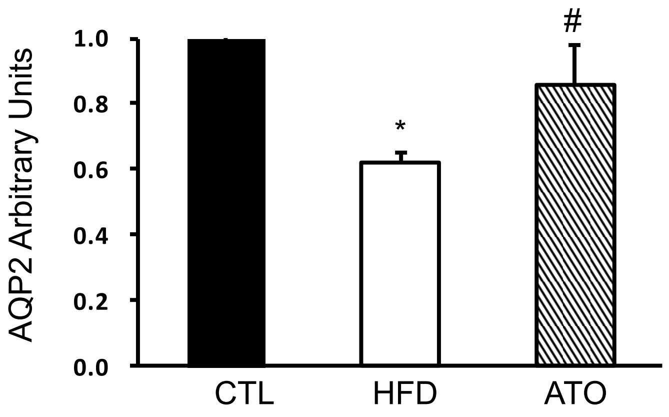

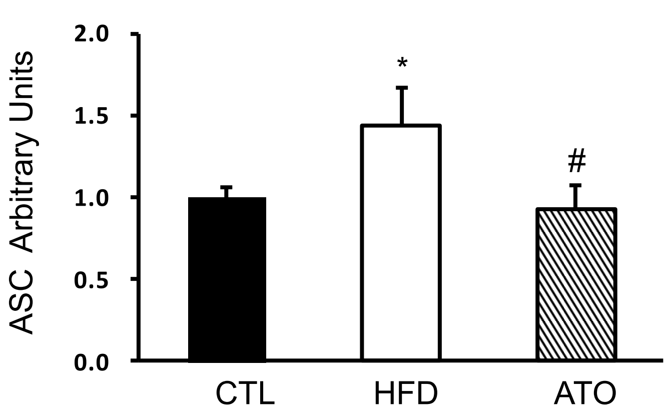

Supplementary data: S6

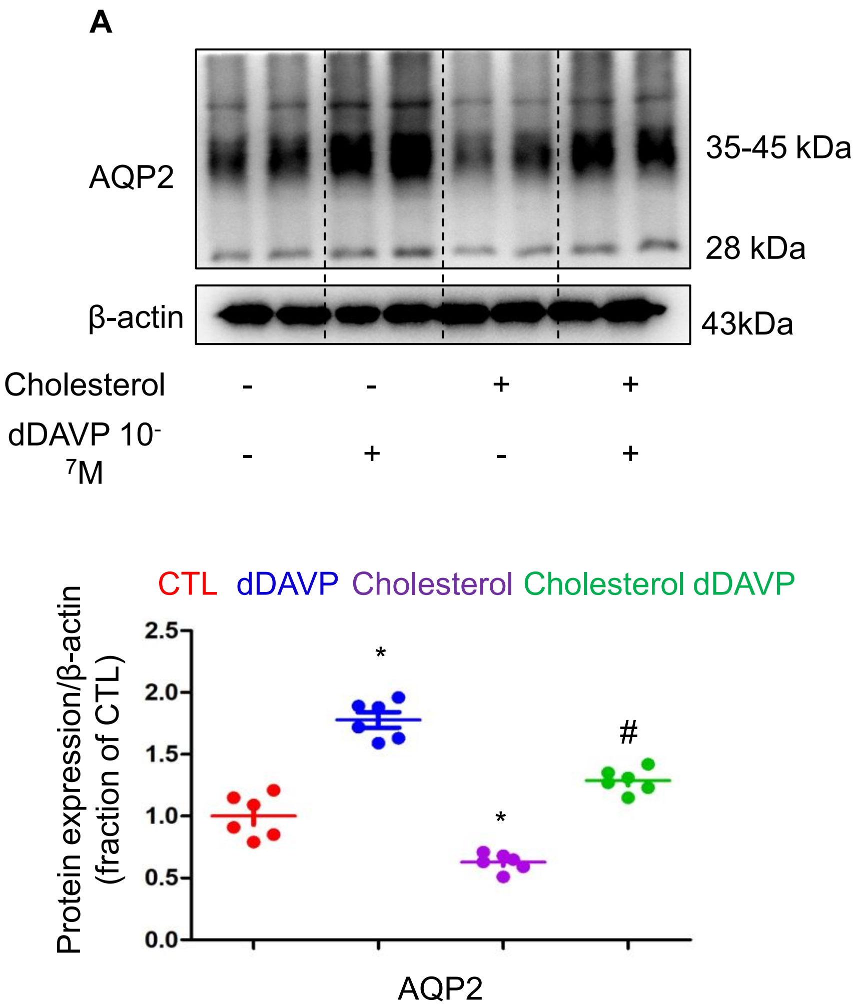

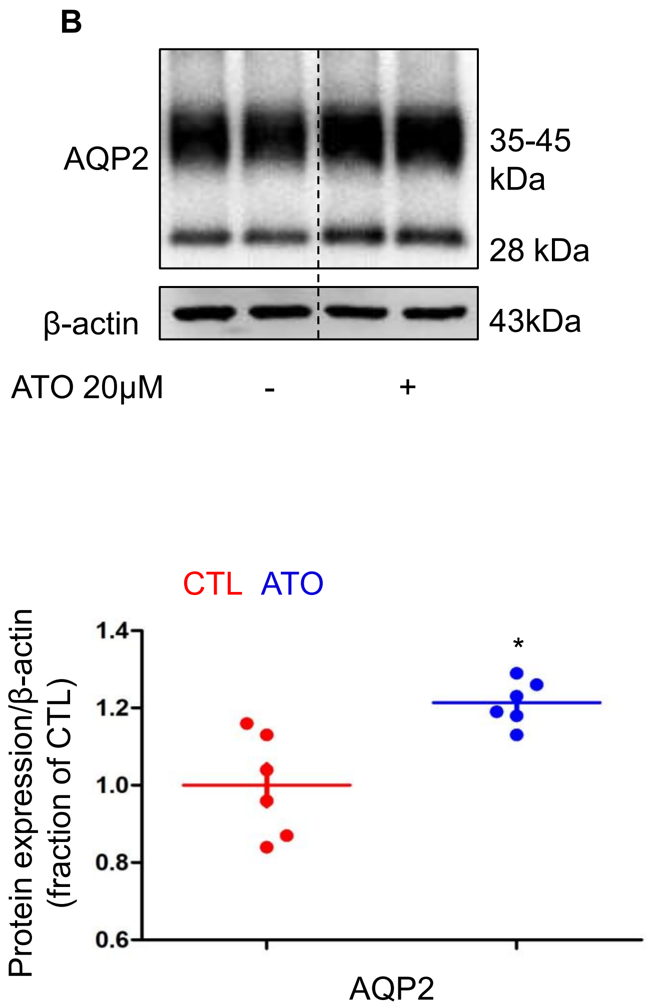

Supplementary data: S7

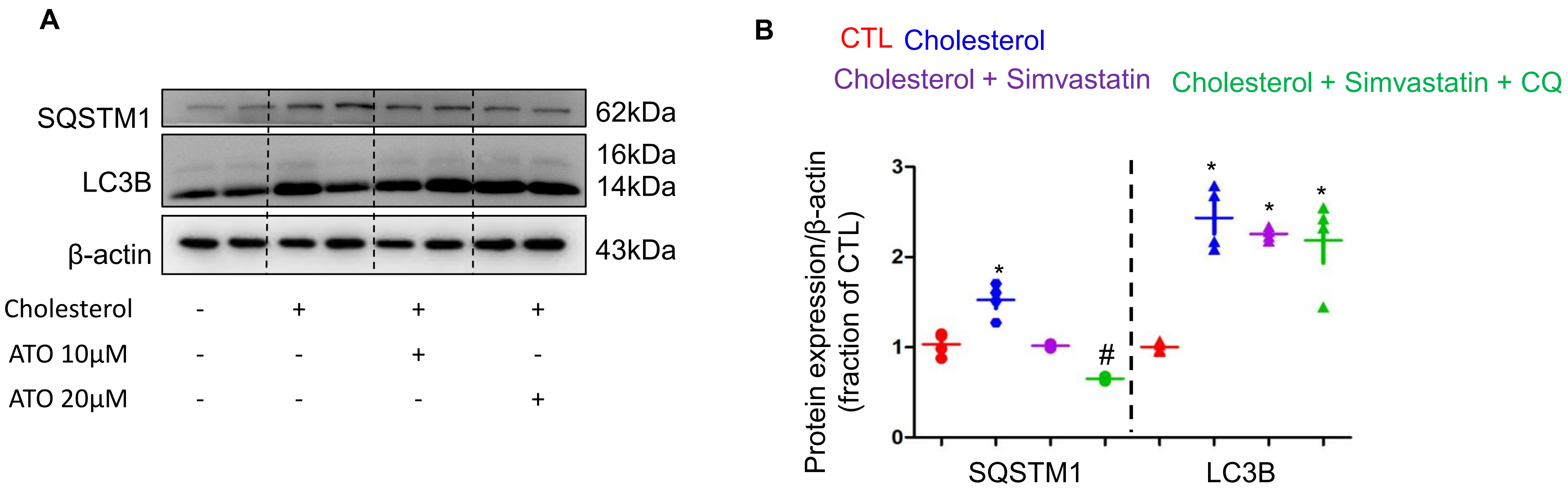

Supplement: Supplementary file 1 — Supplementary figures and tables. [file thnov10p10415s1.pdf]
